# Structure of face-centred icosahedral quasicrystals with cluster close packing

**DOI:** 10.1107/S2053273324008568

**Published:** 2024-09-30

**Authors:** Tsunetomo Yamada, Hiroyuki Takakura, Akiji Yamamoto

**Affiliations:** a Faculty of Advanced Engineering, Department of Applied Physics Tokyo University of Science 6-3-1 Niijuku Katsushika-ku Tokyo 125-8585 Japan; b Division of Applied Physics Faculty of Engineering Hokkaido University Sapporo Hokkaido 060-8628 Japan; c National Institute for Materials Science Tsukuba Ibaraki 305 Japan; Ateneo de Manila University, Philippines

**Keywords:** quasicrystals, higher-dimensional crystallography

## Abstract

A 6D structure model for face-centred icosahedral quasicrystals consisting of so-called pseudo-Mackay and mini-Bergman-type atomic clusters is proposed based on the structure model of the Al_69.1_Pd_22_Cr_2.1_Fe_6.8_ 3/2 cubic approximant crystal (with space group *Pa*3, *a* = 40.5 Å) [Fujita *et al.* (2013). *Acta Cryst*. A**69**, 322–340]. The cluster centres form an icosahedral close sphere packing generated by the occupation domains similar to those in the model proposed by Katz & Gratias [*J. Non-Cryst. Solids* (1993), **153–154**, 187–195], but their size is smaller by a factor τ^2^ [τ = (1 + (5)^1/2^)/2]. The clusters cover approximately 99.46% of the atomic structure, and the cluster arrangement exhibits 15 and 19 different local configurations, respectively, for the pseudo-Mackay and mini-Bergman-type clusters. The occupation domains that generate cluster shells are modelled and discussed in terms of structural disorder and local reorganization of the cluster arrangements (phason flip).

## Abstract 2

Using higher-dimensional crystallography, a new 6D structure model is proposed for *F*-type icosahedral quasicrystals with cluster close packing.

## 1. Introduction

Icosahedral quasicrystals (*i*-QCs) are quasi-periodic long-range ordered crystals that exhibit icosahedral symmetry in their diffraction patterns. Since the discovery of the first *i*-QC in a rapidly cooled alloy of Al–Mn (Shechtman *et al.*, 1984 ▸), many *i*-QCs have been found in various alloy systems. The discovery of face-centred (*F*-type) Al_65_Cu_20_Fe_15_*i*-QC by Tsai *et al.* (1987 ▸) is a milestone in the early stage of QC research, which was followed by the discovery of *F*-type Al_65_Cu_20_*M*_15_ (*M* = Ru or Os) (Tsai *et al.*, 1988 ▸) and Al_70_Pd_20_*M*_10_ (*M* = Mn, Re) *i*-QCs (Tsai *et al.*, 1990 ▸). Because single-grained crystals of these *i*-QCs can be grown with a high degree of crystal perfection, scientists have made a tremendous effort to reveal their atomic structure by single-crystal diffraction and structure modelling (Boudard *et al.*, 1991 ▸; Cornier-Quiquandon *et al.*, 1991 ▸; Katz & Gratias, 1993 ▸; de Boissieu *et al.*, 1994*a* ▸; Gratias *et al.*, 2000 ▸; Yamamoto *et al.*, 2003 ▸, 2004*a* ▸,*b* ▸; Quiquandon & Gratias, 2006 ▸). However, the detailed atomic structure of these *i*-QCs remains an open question.

The atomic structure of QCs can be described in the framework of higher-dimensional crystallography [see, for instance, Yamamoto (1996 ▸), Janssen *et al.* (2007 ▸)]. In the case of *i*-QCs, the atomic structure is described as a 3D section of a 6D periodic structure. The 6D space is defined as a direct sum of two 3D subspaces, the parallel space (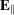) and the perpendicular space (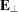), and the 6D structure is described as a periodic arrangement of 3D objects called *occupation domains* (ODs), which are defined in the 3D 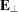. In the analysis of the structure of QCs, various structural parameters are determined by the least-squares method using diffraction intensities. However, the geometrical shape of the ODs has to be determined beforehand and remain unchanged throughout the refinement. This means that the structure analysis is based on a deterministic model; therefore, the construction of a reasonable higher-dimensional model is crucial to a successful structure analysis. An especially successful example is the structure analysis of the binary primitive (*P*-type) Cd_5.7_Yb *i*-QC (Takakura *et al.*, 2007 ▸).

Approximant crystals (ACs) are periodic crystals that exhibit local structures similar to the corresponding QCs (Elser & Henley, 1985 ▸); therefore, knowledge of the atomic structure of the ACs is of great importance in the construction of a higher-dimensional model of the QCs. An AC for an *i*-QC is obtained by introducing a shear strain on the 6D structure of the *i*-QC along the 3D 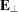 [see, for instance, Quiquandon *et al.* (1999 ▸) and references therein]. A cubic (*c*-) AC attached to a rational number, *q*/*p*, which belongs to the sequence, 1/0, 1/1, 2/1, …, *F*_*n*_/*F*_*n*−1_, which converges to the golden mean, τ = (1 + (5)^1/2^)/2, is sometimes called a Fibonacci AC, where *F*_*n*_ stands for the *n*th Fibonacci number. The lattice constant of *q*/*pc*-AC, *a*_*q*/*p*_, and that of the corresponding *i*-QC, *a*, are connected by the following relation (Elser & Henley, 1985 ▸): 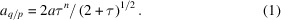
Therefore, if *a*_*q*/*p*_ is known, *a* can be specified, despite the indeterminacy of the lattice constant due to τ^3^- and τ-scaling properties of *P*-type and *F*-type *i*-QCs, respectively.

**Figure 1:**
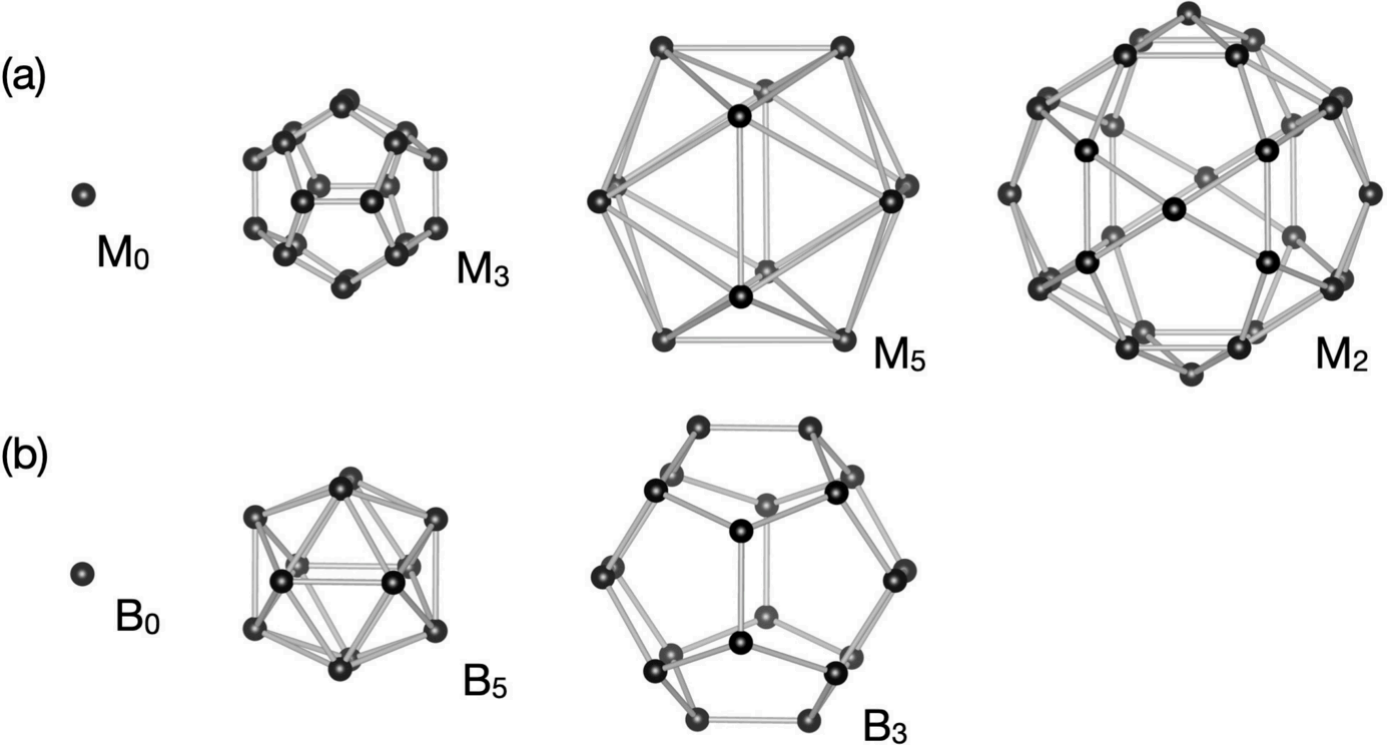
The shell structure of (*a*) pM and (*b*) mB clusters in the Al_69.1_Pd_22_Cr_2.1_Fe_6.8_ 3/2 *c*-AC (Fujita *et al.*, 2013 ▸).

Recently, Fujita *et al.* (2013 ▸) analysed the atomic structure of Al_69.1_Pd_22_Cr_2.1_Fe_6.8_ intermetallic compound (space group 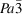, unit-cell parameter 40.5 Å) and revealed that it corresponds to a 2 × 2 × 2 superstructure of Henley’s 3/2 *c*-AC (Henley, 1991 ▸) of *F*-type Al-based *i*-QCs. The lattice constant *a* derived according to equation (1) is approximately 2.8 Å, which is τ times smaller than that of most of the *F*-type Al-based *i*-QCs analysed previously, *e.g.a* = 4.465 Å for Al–Cu–Fe (Cornier-Quiquandon *et al.*, 1991 ▸) and *a* = 4.461 Å for Al–Pd–Mn (Boudard *et al.*, 1992 ▸). Furthermore, it was revealed that the structure consists of two types of atomic clusters, the so-called pseudo-Mackay-type (pM) and mini-Bergman-type (mB) atomic clusters (see Fig. 1 ▸). The former consists of (in order from the centre) a centre (M_0_), a dodecahedron shell (M_3_), a combination of an icosidodecahedron (M_2_) and icosahedron shells (M_5_), while the latter consists of a centre (B_0_), an icosahedron shell (B_5_) and a dodecahedron shell (B_3_). The shell symbols follow the paper by Fujita *et al.* (2013 ▸). The same atomic clusters were considered in the previous structure modellings of *F*-type *i*-QCs (Katz & Gratias, 1993 ▸; Elser, 1996 ▸; Gratias *et al.*, 2000 ▸).

The 3/2 *c*-AC exhibits occupational and positional disorder on the M_3_ and M_2_ shells, and the structure analysis revealed that only specific site classes exhibit structural disorder (Fujita *et al.*, 2013 ▸). Furthermore, the disorder corresponds to tile flip (or phason flip) involving atomic motions on the M_3_ and M_2_ shells in a random tiling model proposed by Elser (1996 ▸). Thus, any 6D model that can take into account this disorder is desirable to analyse the atomic structure of *F*-type *i*-QCs composed of pM and mB clusters in detail.

In this paper, we propose a new 6D model for the *F*-type *i*-QCs that incorporates the findings from the 3/2 *c*-AC. Compared with the model proposed by Katz & Gratias (1993 ▸) (hereafter, referred to as the KG model) and its derivatives, the present model achieves a higher cluster density.

In the following, we use a coordinate system with the proper lattice constant *a* ≃ 2.8 Å based on the lattice constant of the 3/2 *c*-AC and equation (1). The KG model is also described in this coordinate system to facilitate comparison with the present model. The ODs in the present model are subdivided according to all site classes existing on the cluster shells for a precise description of the structural disorder on the M_3_ and M_2_ shells. The construction and subdivision of ODs are performed using a Python library *PyQCstrc.ico* (Yamada, 2021 ▸).

This article is arranged as follows. In Section 2 we briefly introduce the atomic structure of the Al_69.1_Pd_22_Cr_2.1_Fe_6.8_ 3/2 *c*-AC. In Section 3 we present the underlying concepts in the higher-dimensional models of *i*-QCs. In Section 4 we present the new 6D model for the *F*-type *i*-QC constructed based on the 3/2 *c*-AC. In Section 5 we compare the 6D model with existing models of the same structure type. In the last section, we summarize this study.

## 2. Atomic structure of Al_69.1_Pd_22_Cr_2.1_Fe_6.8_ 3/2 *c*-AC

For building and understanding a model consisting of pM and mB clusters, it is important to consider cluster linkage and atomic site classes in shells of a cluster introduced in the 3/2 *c*-AC (Fujita *et al.*, 2013 ▸). They can be briefly summarized as follows.

### 2.1. Shell structure and linkages of pM and mB clusters

**Figure 2:**
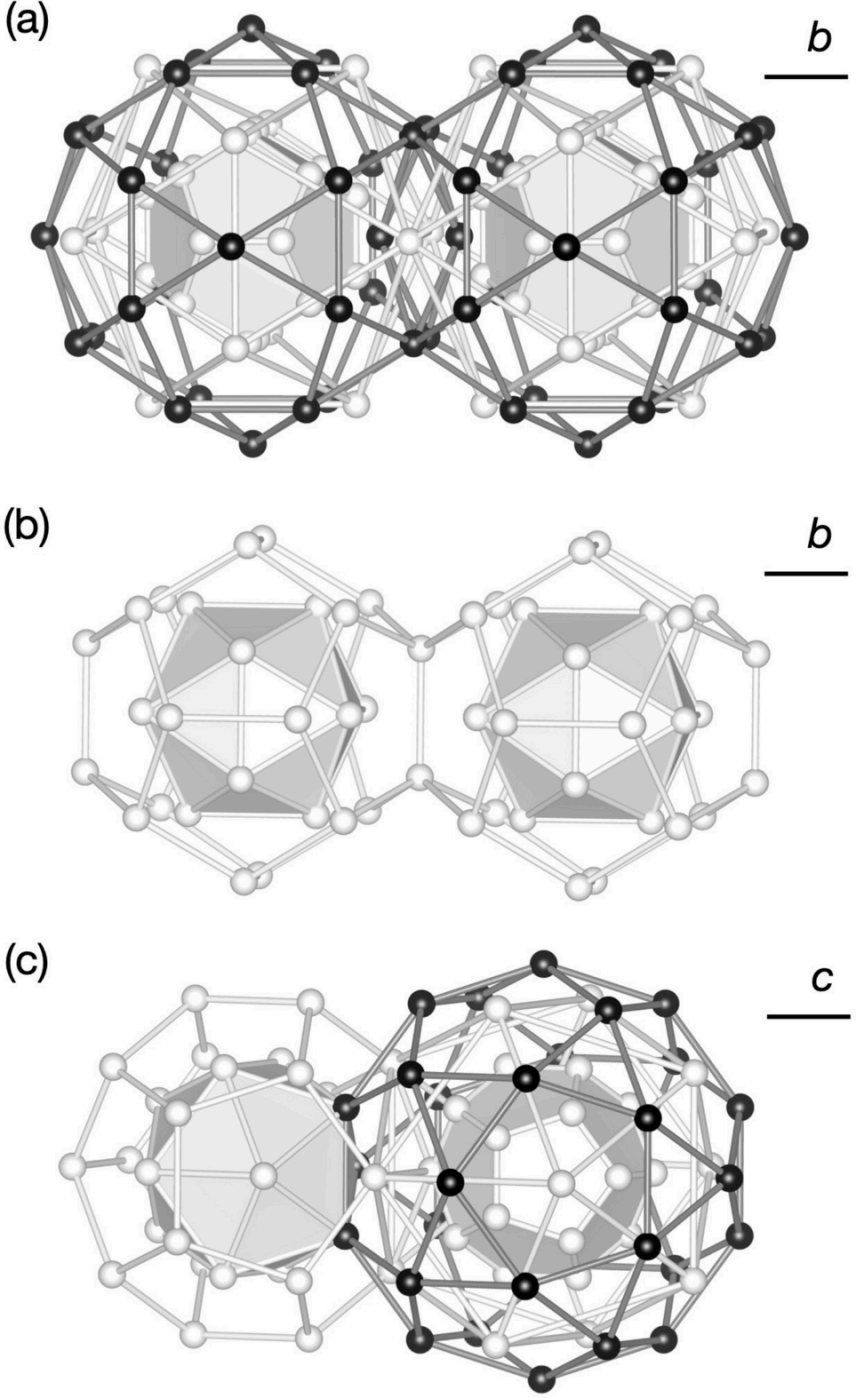
Three types of cluster linkage in the Al_69.1_Pd_22_Cr_2.1_Fe_6.8_ 3/2 *c*-AC (Fujita *et al.*, 2013 ▸): (*a*) *b*-linkage of two pM clusters, (*b*) *b*-linkage of two mB clusters, (*c*) *c*-linkage of mB and pM clusters.

In the 3/2 *c*-AC, a cluster is connected to a neighbouring cluster along a direction parallel to either a twofold or a threefold axis. Hereafter, we call these *b*- and *c*-linkages, respectively, after Henley (1986 ▸). In the former, two clusters of the same kind are connected (pM–pM or mB–mB) with a distance between the two cluster centres of approximately 7.7 Å, while in the latter pM and mB clusters are connected with a distance between them of approximately 6.7 Å, as depicted in Fig. 2 ▸ (Fujita *et al.*, 2013 ▸). With these linkages, the cluster shells touch or intersect each other by sharing some atomic sites as follows.

In a *b*-linkage of pM clusters, the clusters intersect each other and share four sites of the M_2_ shell and two sites of the M_5_ shell [see Fig. 2 ▸(*a*)]. The common part forms a flat hexagonal bipyramid, and two M_2_ sites of the bipyramid located on the linkage are close to each other (approximately 1.8 Å).

Because of the short distance, these M_2_ sites cannot be occupied by two atoms at the same time; therefore, these sites must be considered as a splitting site with an occupation probability equal to 1/2. In a *b*-linkage of mB clusters, the B_3_ shell shares an edge and two sites on the shell [see Fig. 2 ▸(*b*)]. In a *c*-linkage, pM and mB clusters interpenetrate heavily, and the sites of the M_2_ and M_5_ shells are, respectively, shared with the sites of the B_5_ and B_3_ shells [see Fig. 2 ▸(*c*)]. Furthermore, one site of the B_3_ shell is situated within the pM cluster and shares with a site of the inner M_3_ shell.

### 2.2. Site class

In the cluster packing with the *b*- and *c*-linkages, most of the atomic sites are shared by more than one cluster shell. Fujita *et al.* (2013 ▸) characterized the atomic sites in the 3/2 *c*-AC according to ‘site class’: if an atomic site belongs to *n* cluster shells, X^1^, X^2^, …, X^*n*^, then the class associated with this site is expressed by 〈X^1^, X^2^, …, X^*n*^〉. In total, 16 different classes exist in the 3/2 *c*-AC, and the authors pointed out that positional and occupational disorder are present at the atomic sites that belong to specific classes.

The former is related to the atomic site represented by 〈M_2_〉, which corresponds to the splitting sites on the pM clusters. The latter is related to the M_3_ site, which belongs to 〈M_3_, B_3_〉 or 〈M_3_〉. The 〈M_3_, B_3_〉 represents the atomic site that shares the M_3_ and B_3_ shells on the *c*-linkage, while the 〈M_3_〉 represents the rest of the M_3_ site. The structure analysis showed that the M_3_ site represented by 〈M_3_, B_3_〉 is occupied, but that represented by 〈M_3_〉 is vacant (Fujita *et al.*, 2013 ▸). The positional and occupational disorder are supposed to exist in the atomic structure of the corresponding *F*-type *i*-QCs; therefore, site classes existing in the *i*-QCs will be useful to analyse the atomic structure in more detail.

## 3. Underlying concepts for the 6D model of *i*-QCs

To consider the arrangement of pM and mB clusters in the present model and the relation to the KG model described in different coordinate systems, the similarity transformation plays an important role. In this section, model descriptions in different but equivalent coordinate systems are presented.

### 3.1. 3D Amman–Kramer–Neri tiling

**Figure 3:**
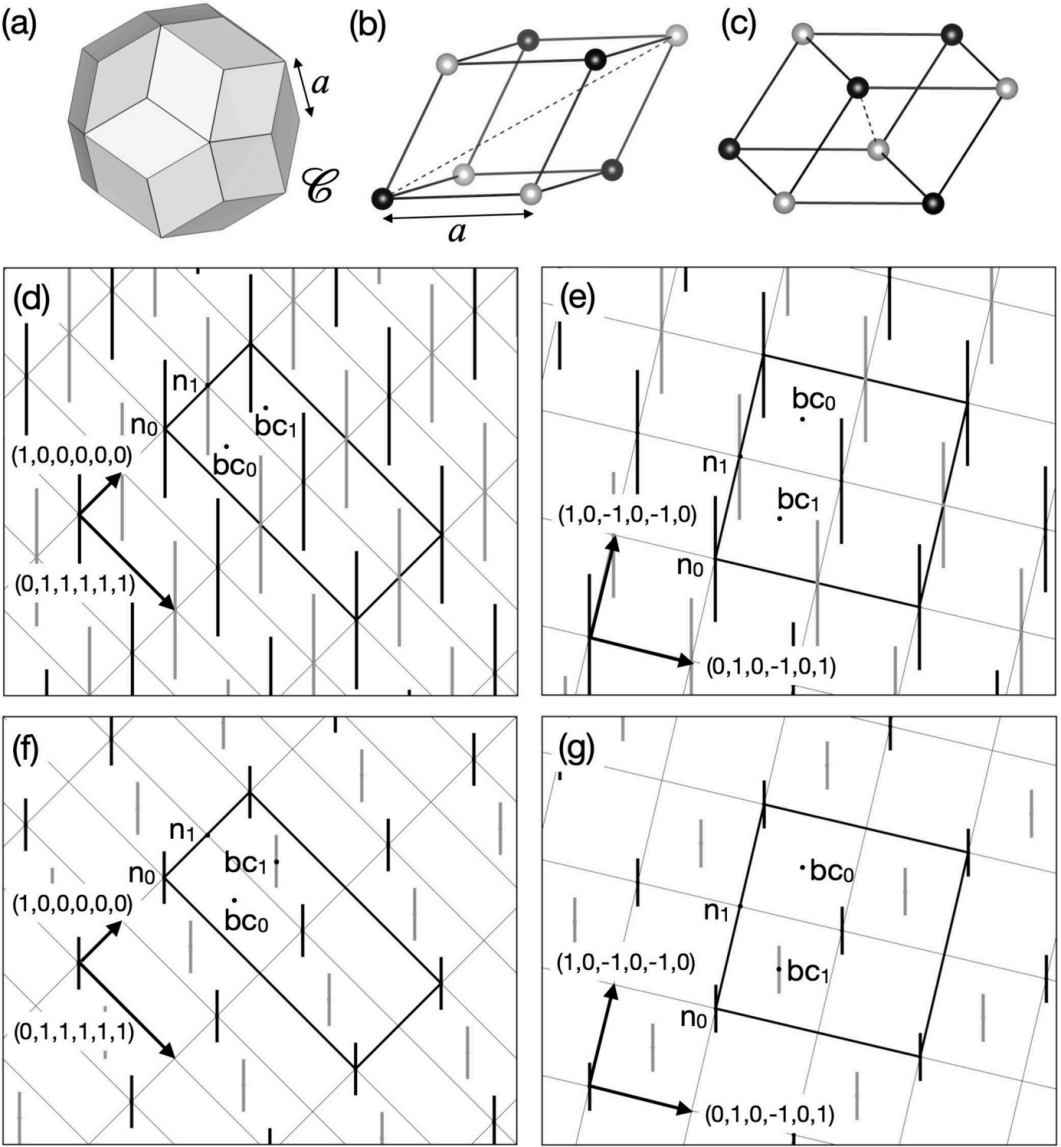
Relationship between the *F*-type 3D AKN tiling with edge length of *a* and that with edge length of τ^2^*a*. (*a*) Rhombic triacontahedron OD 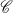 with edge length of *a*, (*b*) acute rhombohedron and (*c*) obtuse rhombohedron units in the former tiling. The black and grey balls correspond to ‘even’ and ‘odd’ vertices, respectively. The dashed line represents the threefold axis of each rhombohedron. The arrangement of 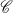
’s on (*d*) the fivefold plane spanned by (1, 0, 0, 0, 0, 0) and (0, 1, 1, 1, 1, 1), (*e*) the threefold plane spanned by 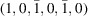 and 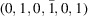 in the *F*-type 6D superstructure that generates the *F*-type 3D AKN tiling with edge length of *a*. The arrangement of 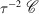
’s, which generates the *F*-type AKN tiling inflated by τ^2^, in (*f*), (*g*), is obtained by the similarity transformation of order 2 from that of 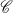
’s in (*d*), (*e*). The black and grey line segments in (*d*)–(*g*) represent the 2D sections of 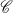
’s or 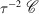
’s that generate ‘even’ and ‘odd’ vertices, respectively. The thick black line shows the 2D section of the 6D unit cell.

The 3D Amman–Kramer–Neri (AKN) tiling is known as a simple icosahedral tiling, and it consists of two building units, *i.e.* acute and obtuse rhombohedra (Kramer & Neri, 1984 ▸; Duneau & Katz, 1985 ▸; Elser, 1985 ▸, 1986 ▸; Levine & Steinhardt, 1986 ▸; Henley, 1986 ▸). The vertices of the tiling with an edge length of *a* are obtained from a *P*-type 6D structure. This is described as a periodic arrangement of a rhombic triacontahedron (RT) OD with an edge length of *a*, denoted as 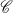 [Fig. 3 ▸(*a*)], located at either the vertex (0, 0, 0, 0, 0, 0) or body-centre (1, 1, 1, 1, 1, 1)/2 position in the unit cell of a *P*-type 6D lattice. Unit vectors of the 6D lattice used in this paper can be found in Appendix *A*[App appa].

We consider an *F*-type 6D superstructure with a doubled lattice constant on the *P*-type lattice (Rzepski *et al.*, 1989 ▸). The *F*-type lattice has four special positions with site symmetry of 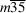 and their 6D coordinates with respect to the underlying *P*-type lattice are vertex (0, 0, 0, 0, 0, 0), edge-centre (1, 0, 0, 0, 0, 0), and two independent body-centre positions (1, 1, 1, 1, 1, 1)/2 and (3, 1, 1, 1, 1, 1)/2. In the following, we denote these positions as n_0_, n_1_, bc_0_ and bc_1_, respectively, after Boudard *et al.* (1992 ▸).

The 3D AKN tiling with the edge length of *a* is described in an *F*-type 6D lattice as well. It is obtained from 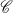 situated at both n_0_ and n_1_ positions, as shown in Figs. 3 ▸(*a*)–3 ▸(*e*). Since the *F*-type lattice has a self-similarity with similarity ratio τ, the AKN tiling with an edge length of τ^*m*^*a* for arbitrary integer *m* (order *m* hereafter) is describable in the same lattice but the length of the edge of the RT is smaller by a factor τ^*m*^ (see Appendix *B*[App appb]).

Let us consider two AKN tilings inflated by τ and τ^2^ which are useful to understand the KG model described in the current coordinate system and the location of cluster centres. The AKN tiling inflated by τ is obtained by the similarity transformation of order 1 from the original one. Under this similarity transformation, positions n_0_, n_1_, bc_0_ and bc_1_ are transformed to n_0_, bc_1_, bc_0_ and n_1_, respectively, while the size of the ODs is deflated by a factor τ^−1^. The AKN tiling inflated by τ^2^ is obtained by applying this transformation again. See Figs. 3 ▸(*f*) and 3 ▸(*g*), where ‘even’ and ‘odd’ vertices of an inflated tiling with an edge length of τ^2^*a* are generated from the ODs deflated by a factor τ^−2^ at n_0_ and bc_1_, respectively.

**Figure 4:**
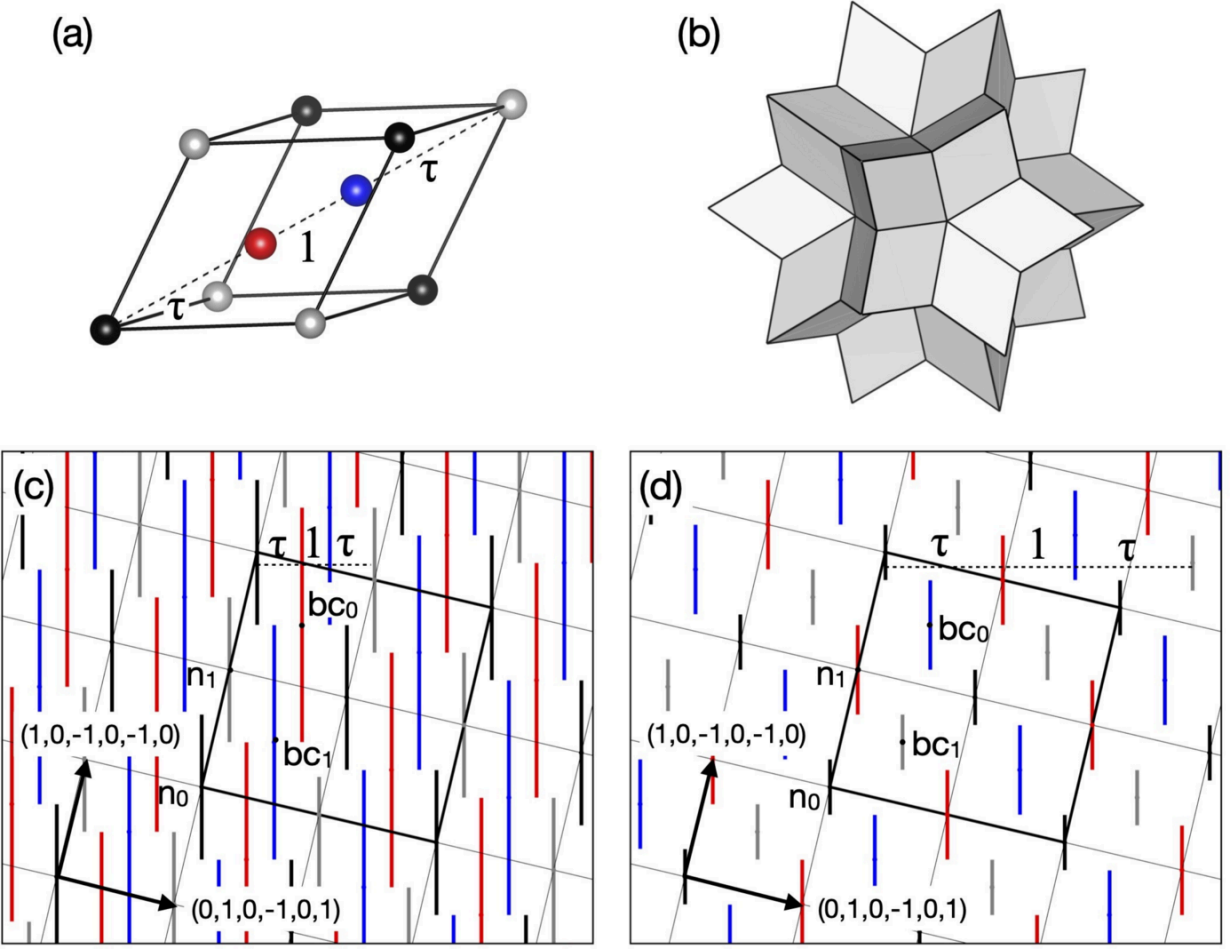
Relationship between the decorated *F*-type 3D AKN tiling with edge length of *a* and that with edge length of τ^2^*a*. (*a*) Acute rhombohedron decorated with two positions that divide the longer body-diagonal by τ:1:τ. (*b*) Dodecahedral star OD used for the decoration. (*c*) 6D structure of the decorated *F*-type 3D AKN tiling with edge length of *a*, which is obtained by adding dodecahedral star ODs with edge length of *a*, at bc_0_ and bc_1_, to the 6D superstructure in Fig. 3 ▸(*e*). The red and blue line segments represent the 2D sections of the dodecahedral star ODs, which generate the two positions represented by the red and blue balls in (*a*), respectively. (*d*) 6D structure of the decorated *F*-type 3D AKN tiling with edge length of τ^2^*a* obtained by the similarity transformation of order 2 from (*c*). The thick black squares in (*c*), (*d*) indicate the 2D sections of the 6D unit cell.

The rhombohedral units in the *F*-type 3D AKN tiling can be decorated by placing additional ODs onto the corresponding 6D structure. For example, the two positions that divide the longer body-diagonals of all acute rhombohedra of the *F*-type 3D AKN tiling with edge length *a* into τ:1:τ [Fig. 4 ▸(*a*)] are generated by placing the two dodecahedral star ODs with edge length *a* [Fig. 4 ▸(*b*)] at bc_0_ and bc_1_. On the other hand, in the same coordinate system, the two positions in all acute rhombohedra of the *F*-type 3D AKN tiling with edge length of τ^2^*a* are generated by two dodecahedral star ODs with edge length τ^−2^*a* placed at n_1_ and bc_0_. Their 6D structures are shown in Figs. 4 ▸(*c*) and 4 ▸(*d*), respectively. In particular, the latter is related to the location of pM clusters in the present model, as will be shown later.

### 3.2. Katz and Gratias’s model

**Figure 5:**
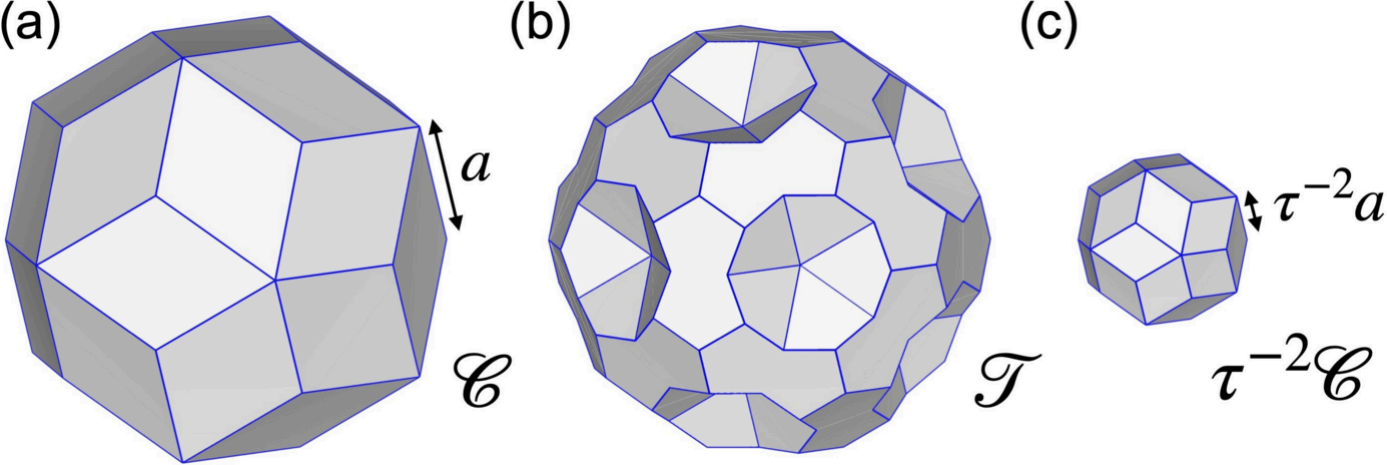
Three ODs comprising the scaled-KG model. (*a*) 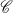 at bc_0_, (*b*) 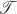 at n_0_ and (*c*) 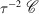 at bc_1_. Note that these ODs are obtained by the similarity transformation of order 1 from the original model by Katz & Gratias (1993 ▸) after shifting the origin to bc_0_.

The 6D model for the *F*-type *i*-QCs proposed by Katz & Gratias (1993 ▸) and Cockayne *et al.* (1993 ▸) is described by the 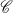, 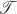 and 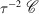 located at bc_0_, n_0_ and bc_1_, respectively (see Fig. 5 ▸) in the current coordinate system. This transformed KG model (henceforth referred to as the ‘scaled-KG model’) is obtained by applying a similarity transformation of order 1 and an origin shift of (1, 1, 1, 1, 1, 1)/2 to the original model, which consists of three ODs 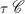, 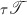 and 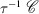 located at n_0_, n_1_ and bc_1_, respectively, with a lattice constant of τ*a*.

The volume of the asymmetric unit of the 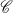, 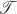 and 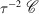 in the scaled-KG model is given as (1 + 2τ)/6, (−3 + 4τ)/6 and (−3 + 2τ)/6 in a unit of (2 + τ)^−3/2^, respectively. In the following, we use this unit to simplify the expression of 3D volumes of ODs.

## 4. 6D Model of the *F*-type *i*-QCs consisting of pM and mB clusters

In a cluster-based model of QCs, the location of the cluster has to be determined first. In the present model, cluster centres that form a close-packed icosahedral symmetry are considered. As shown by Quiquandon *et al.* (2014 ▸), the ODs in the KG model form an icosahedral close sphere packing. This means that their transformed ODs by similarity transformation of order *m* form a close sphere packing with a τ^*m*^-scaled sphere radius. In the following, we consider ODs giving the cluster positions which have the same *b*- and *c*-linkages as those in the 3/2 *c*-AC and form an icosahedral close-packed distribution.

### 4.1. Cluster centres

**Figure 6:**
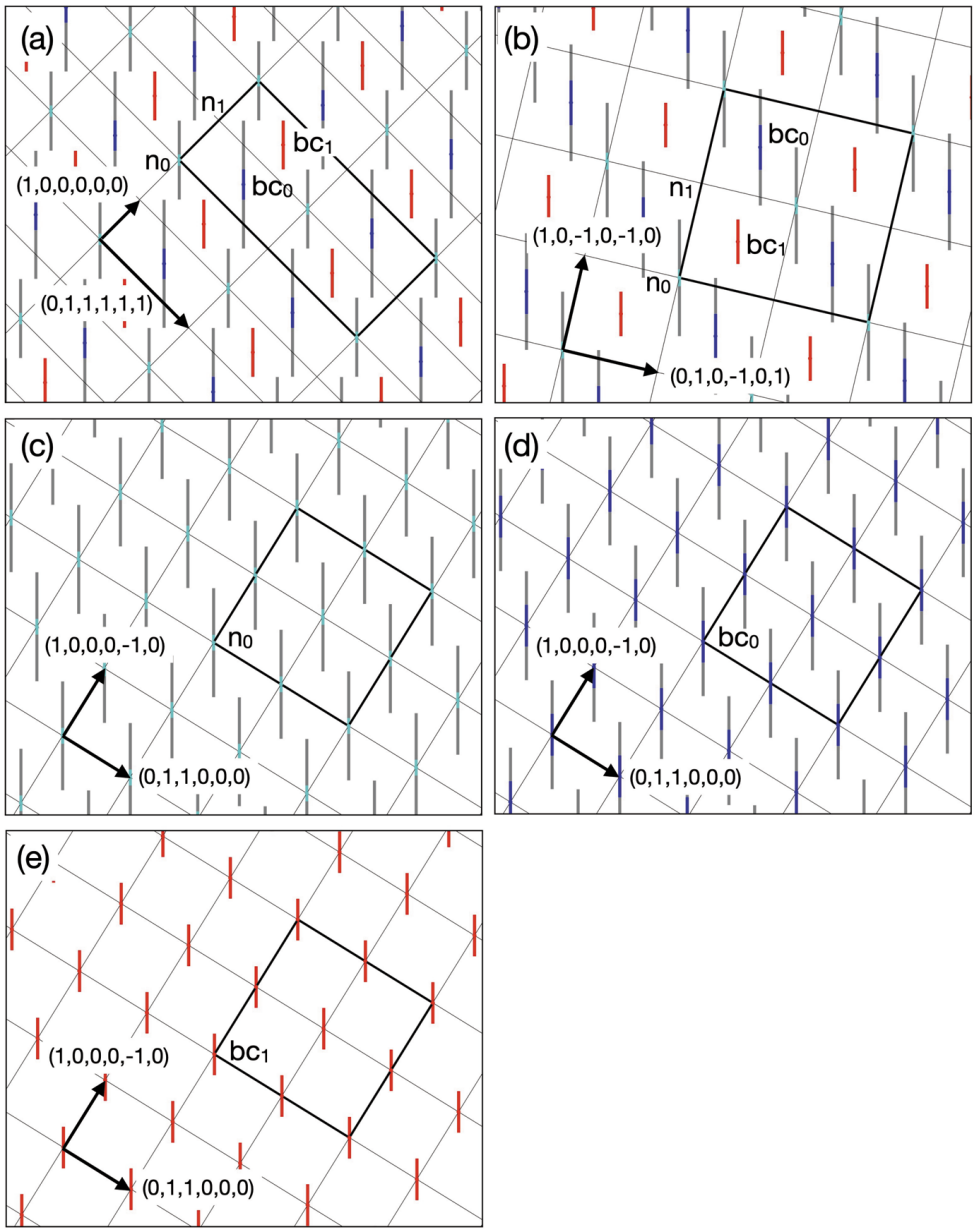
Arrangement of ODs for the cluster centres on the 6D lattice. (*a*) Fivefold plane spanned by (1, 0, 0, 0, 0, 0) and (0, 1, 1, 1, 1, 1), (*b*) threefold plane spanned by 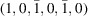 and 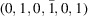, and (*c*)–(*e*) twofold planes spanned by 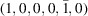 and (0, 1, 1, 0, 0, 0). The twofold planes pass through (*c*) n_0_, (*d*) bc_0_ and (*e*) bc_1_. Line segments in blue, cyan and red represent 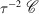 at bc_1_, 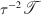 at bc_0_ and 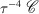 at n_0_, respectively. Line segments in grey represent the three ODs of the scaled-KG model. The thick black square or rectangle shows a 2D section of the 6D unit cell.

In the present 6D model, the positions of cluster centres are generated by three archetype ODs that have the same shape as those of the scaled-KG model (Fig. 5 ▸), but their sizes are τ^−2^ times. These ODs are obtained by the similarity transformation of order 2 from the scaled-KG model. The arrangement of the archetype ODs on the 6D lattice is shown in Fig. 6 ▸. They are arranged as follows: first, the centres of the pM cluster are generated by 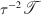 at bc_0_ and 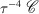 at n_0_; second, those of the mB cluster are generated by 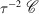 located at bc_1_. Consequently, the ratio of pM to mB clusters in the present model becomes (−21 + 13τ):(−3 + 2τ)/6, namely 46.7%: 53.3%, approximately. These values are close to 48.5% and 51.5% for pM and mB clusters, respectively, in the 3/2 *c*-AC (Fujita *et al.*, 2013 ▸). Hereafter, we distinguish the pM clusters generated by 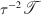 and 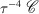 by the notations pM and pM′, respectively.

**Figure 7:**
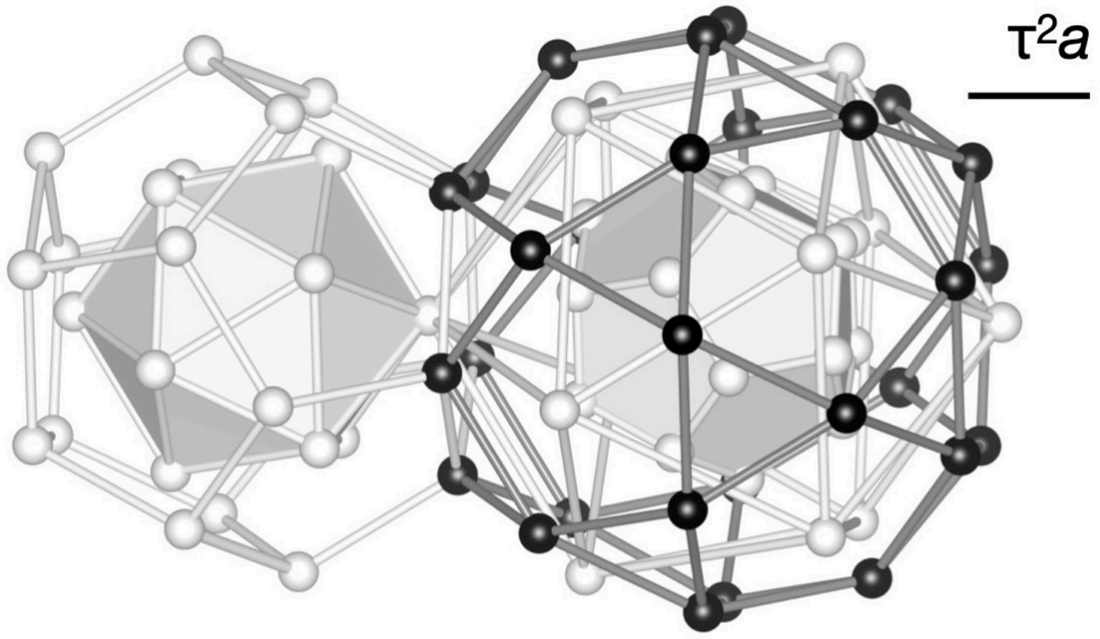
A τ^2^*a*-linkage between mB and pM′ clusters along a fivefold direction.

The cluster-packing geometry resulting from the above 6D model consists of a cluster linkage along a direction parallel to a fivefold axis with a distance of τ^2^*a* between the cluster centres (hereafter referred to as the τ^2^*a*-linkage) (Kitahara & Kimura, 2017 ▸), in addition to the *b*- and *c*-linkages observed in the 3/2 *c*-AC (see Fig. 2 ▸). In the τ^2^*a*-linkage, different kinds of clusters (pM′–mB) are connected to each other along the direction parallel to a fivefold axis (see Fig. 7 ▸). With this linkage, a pentagonal facet of the B_3_ and M′_2_ shells is shared, and one site on the B_5_ and M′_5_ shells is shared. In the *b*-linkage, the same kind of clusters (pM–pM or mB–mB) are connected with a distance of *b* = *a*(4 + 8/(5)^1/2^)^1/2^ between the two cluster centres, while the *c*-linkage of the pM and mB clusters has a distance of *c* = *b*((3)^1/2^/2).

When comparing the ODs for cluster centres in the present model [see Fig. 6 ▸(*b*)] and those in the decorated *F*-type 3D AKN tiling with edge length of τ^2^*a* [see Fig 4 ▸(*d*)], we find the following correspondences: first, 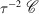 at bc_1_ for the mB cluster centres is identical to the OD that generates ‘odd’ (bc_1_) vertices of the AKN tiling; second, 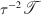 at bc_0_ for the pM cluster centres corresponds to the dodecagonal star OD with edge length of τ^−2^*a*, although the former is part of the latter [see Fig 4 ▸(*d*)]; third, 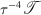 at n_0_ for pM′ cluster centres corresponds to the OD that generates ‘even’ (n_0_) vertices of the AKN tiling, although the former is τ^−2^ times the latter. The first means that the mB clusters are located at all the ‘odd’ vertices of the tiling. The second means that the pM clusters are located at some of the body-diagonal positions in the acute rhombohedra. Similarly, the third indicates that the pM′ clusters are located at some of the ‘even’ vertices of the tiling. The precise position of the pM+pM′ cluster centres is described in the following.

**Figure 8:**
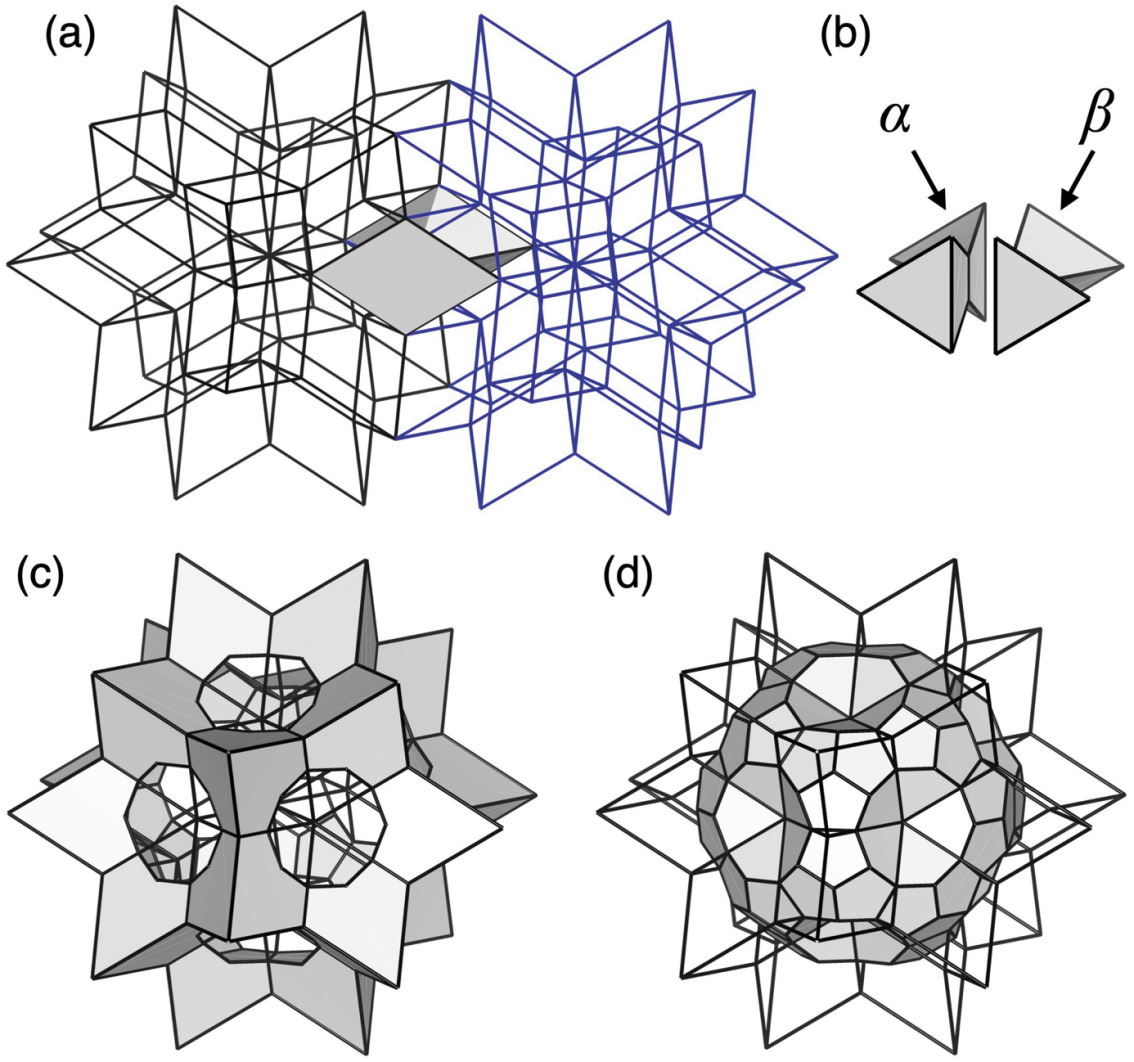
Decomposition of the dodecahedral star OD with the edge length of τ^−2^*a*. (*a*) An intersection of two dodecahedral stars. The black and blue domains are located at (1, 1, 1, 1, 1, 1)/2 and (1, 3, 3, 1, 1, 1)/2, respectively. The common part is highlighted in grey. (*b*) A decomposition of the common part in (*a*). The ODs indicated by α and β have the same volume. (*c*) Union of a domain indicated by β and its equivalents under the icosahedral symmetry at (1, 1, 1, 1, 1, 1)/2. (*d*) The OD obtained by removing that in (*c*) from the dodecahedral star.

Because the dodecahedral star OD, having edge length of τ^−2^*a*, at bc_0_ partially intersects its copies shifted by (0, 1, 1, 0, 0, 0) and its equivalents, it generates pairs of two sites separated by a distance of (τ − 1)*b*/2 along twofold directions. This length is too short to link two pM clusters. Therefore, one of the two sites in each pair has to be removed to realize the same cluster-packing geometry as in the 3/2 *c*-AC. Fig. 8 ▸ shows how the dodecahedral star OD can be decomposed so as to eliminate the short linkages of (τ − 1)*b*/2. Because this linkage is generated by the ODs indicated by α and β, which are the common part of the dodecahedral star OD and its copies shifted by (0, 1, 1, 0, 0, 0), and their equivalents, the union of the ODs indicated by α and β has to be divided into two parts with the same volume, without breaking the icosahedral symmetry. In the present case, we divide into two parts by a plane perpendicular to a twofold axis so that the resulting ODs are those indicated by α and β, and remove the latter from the dodecahedral star OD. The resulting OD is identical to 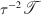 for the pM cluster centres. Together with the uncommon part and α, the resulting shape of the OD becomes identical to 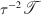. Therefore, such a short linkage of pM clusters does not appear in the present model.

**Figure 9:**
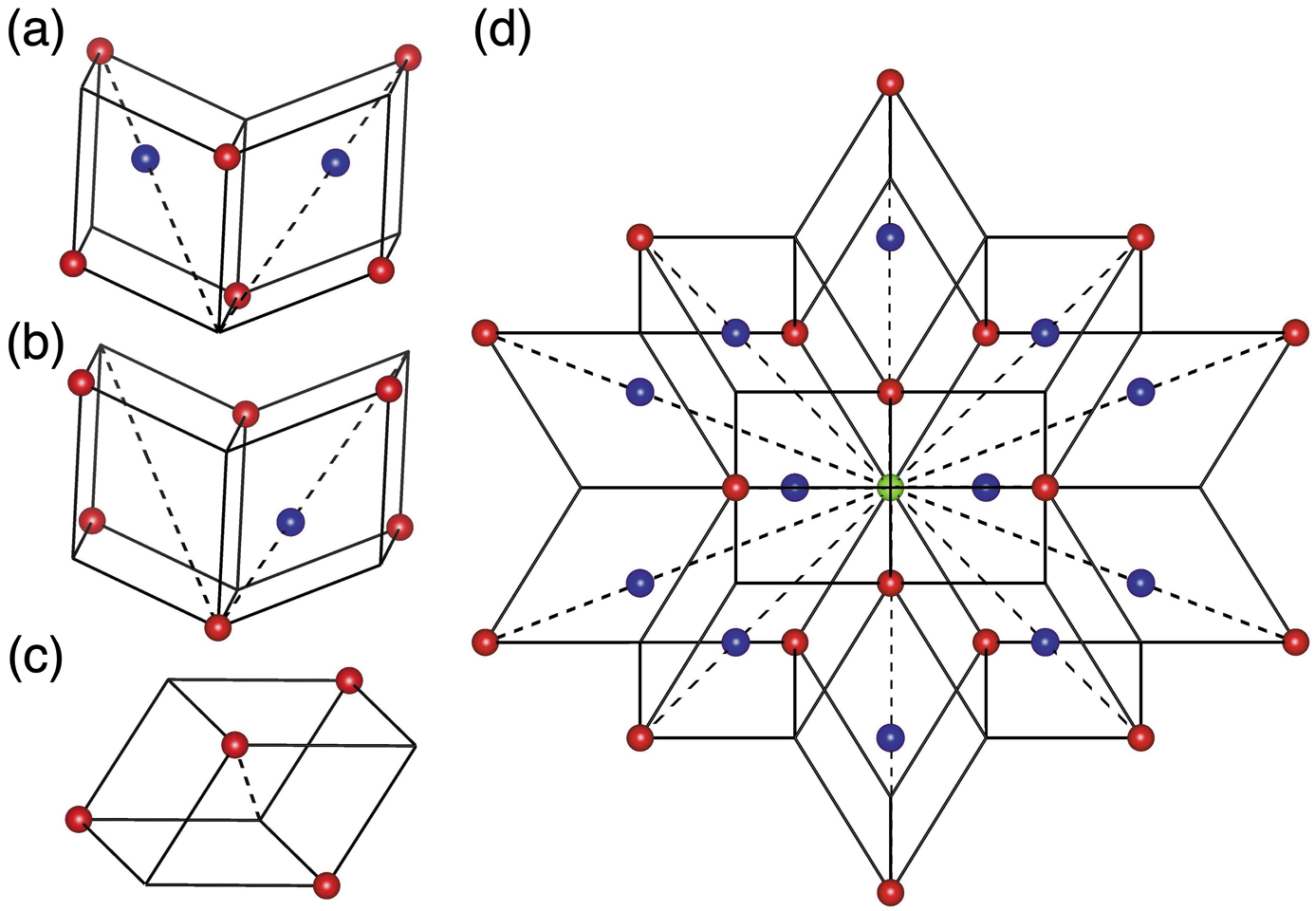
Positions of cluster centres in the *F*-type 3D AKN tiling with edge length of τ^2^*a*. The ‘even’ vertices on the threefold axis are (*a*) shared and (*b*) unshared with the two neighbouring rhombohedra. (*c*) The obtuse rhombohedron. (*d*) The dodecahedral star configuration is composed of 20 acute rhombohedra. The central position corresponds to an ‘even’ 12-fold vertex in the 3D AKN tiling. The blue, green and red balls represent the centre positions of the pM, pM′ and mB clusters, respectively. The mB clusters are located at every ‘odd’ vertex of the tiling. The dashed line represents the threefold axis of each rhombohedron.

Fig. 9 ▸ shows the position of cluster centres in an *F*-type 3D AKN tiling with edge length of τ^2^*a*. When two acute rhombohedra share an ‘even’ vertex on the longer body-diagonal, there is one pM cluster in each rhombohedron, and they are positioned to form a *b*-linkage [see Fig. 9 ▸(*a*)]. In contrast, when two acute rhombohedra share an ‘odd’ vertex on the longer body-diagonal, one rhombohedron has a pM cluster positioned at τ:1 dividing its diagonal from the ‘even’ vertex to ‘odd’ vertex direction, while the other has no pM cluster [see Fig. 9 ▸(*b*)]. In this way, the short inter-cluster distance of (τ − 1)*b*/2 is avoided. The pM clusters are connected to neighbouring mB clusters with *c*-linkages, while the pM′ cluster is situated at the ‘even’ 12-fold vertices of the AKN tiling with edge length of τ^2^*a*. The cluster is surrounded by 12 mB clusters located at ‘odd’ vertices. An example of the 12-fold vertices is depicted in Fig. 9 ▸(*d*) where the central position in a dodecahedral star configuration is composed of 20 acute rhombohedra.

**Figure 10:**
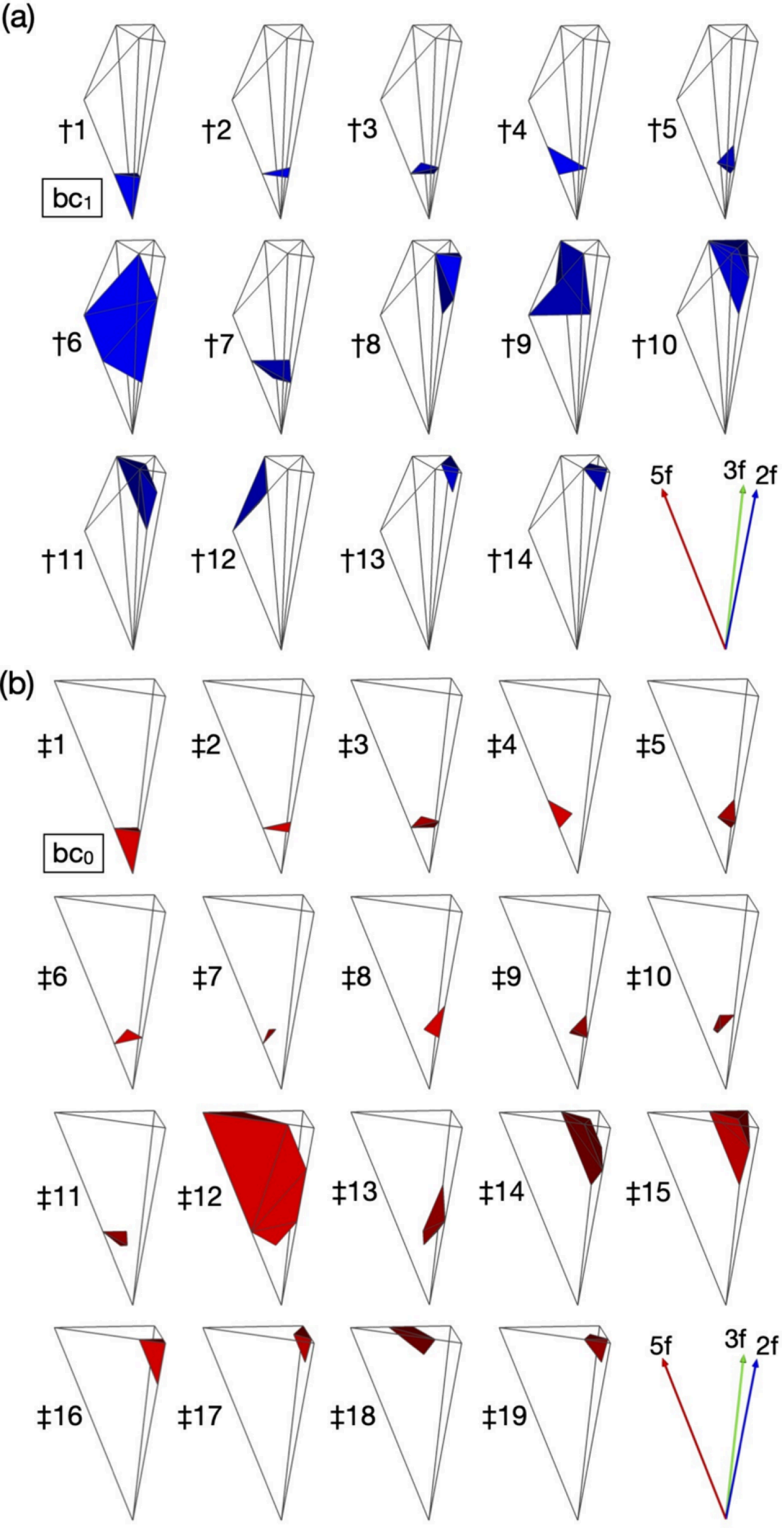
Subdivisions of asymmetric units of 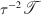 at bc_0_ and 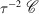 at bc_1_, which generate cluster centre positions M_0_ and B_0_, respectively, according to local configurations for pM (blue) and mB (red) clusters (see Table 1 ▸).

Knowledge of local configurations of the first neighbouring clusters in the *F*-type *i*-QCs is important to analyse disorder sites, because both the occupational disorder on the M_3_ shell and the splitting sites (*i.e.* positional disorder sites) on the M_2_ shell depend on the local configurations of the clusters. Such local configuration is determined by identifying the ODs of cluster centres that intersect each other when projected onto the 3D 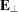 (Gratias *et al.*, 2000 ▸; Duneau & Gratias, 2002 ▸; Takakura, 2008 ▸; Takakura & Strzałka, 2017 ▸). It turns out that there are in total 15 and 19 different configurations for the pM+pM′ and mB clusters in the present model, respectively. The shape of the ODs corresponding to each configuration is determined and their asymmetric units are presented in Fig. 10 ▸. Furthermore, the coordination number (CN) of the first neighbouring clusters and the number of constituting linkages, the volume of the OD and frequency of each local configuration of the pM+pM′ and mB clusters are listed in Table 1 ▸. Hereafter, the configurations are expressed by a notation (CN, Z_*a*_, Z_*b*_, Z_*c*_) after Henley (1986 ▸), where Z_*a*_, Z_*b*_ and Z_*c*_ are the number of τ^2^*a*-, *b*- and *c*-linkages in CN, respectively. The most abundant local configuration of the pM cluster is (12, 0, 5, 7) with a frequency of approximately 19.23%, which corresponds to †6 in Table 1 ▸, and that of the mB cluster is (13, 1, 7, 5) with a frequency of approximately 35.33%, which corresponds to ‡12 in Table 1 ▸. There is only one cluster configuration for the pM′ cluster, *i.e.* the ‘even’ 12-fold vertices, which corresponds to †15 in Table 1 ▸. The configuration is (12, 12, 0, 0): each pM′ cluster is surrounded by 12 mB clusters with the τ^2^*a*-linkages. Here, we note that the local configurations of the ‘clusters’ in Table 1 ▸ are identical to those of the ‘atoms’ derived by Gratias *et al.* (2000 ▸). This is because the ODs for the cluster centres in the present model are obtained by similarity transformation of order 2 from the scaled-KG model.

### 4.2. Cluster shells

In the cluster-based model, ODs for the cluster shells (hereafter, referred to as shell-ODs) are modelled using the ODs that have the same shape and size as the ODs for the cluster centres, namely archetype ODs (Yamamoto & Hiraga, 1988 ▸). The shell-ODs for the pM cluster are derived from the archetype OD 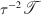, as follows. First, the OD that generates the M_2_ shell is a union of 30 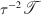, and each OD is shifted by −(0, 1, 1, 0, 0, 0)_⊥_ or its equivalents from bc_0_. Here, the subscript ⊥ denotes the perpendicular-space components of a 6D vector. Second, the OD that generates the M_3_ shell is a union of 20 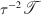 shifted by 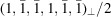 and its equivalents from n_0_. Third, the OD that generates the M_5_shell is a union of 12 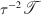, and each OD is shifted by −(1, 1, 1, 1, 1, 1)_⊥_/2 or its equivalents from n_0_.

In the same manner, the shell-ODs for the pM′ cluster are derived from the archetype OD 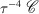 as follows. First, the OD that generates the M_2_′ shell is a union of 30 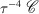, and each OD is shifted by −(0, 1, 1, 0, 0, 0)_⊥_ or its equivalents from n_0_. Second, the OD for the M_3_′ shell is a union of 20 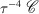 shifted by 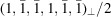 and its equivalents from bc_0_. Third, the OD for the M_5_′ shell is a union of 12 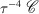, and each OD is shifted by −(1, 1, 1, 1, 1, 1)_⊥_/2 or its equivalents from bc_0_.

Similarly, the shell-ODs for the mB cluster are derived from the archetype OD 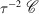 as follows. First, the OD for the B_3_ shell is a union of 20 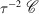, and each OD is shifted by 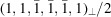 or its equivalents from n_0_. Second, the OD for the B_5_ shell is a union of 12 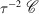 shifted by (1, 0, 0, 0, 0, 0)_⊥_ and its equivalents from bc_0_.

**Figure 11:**
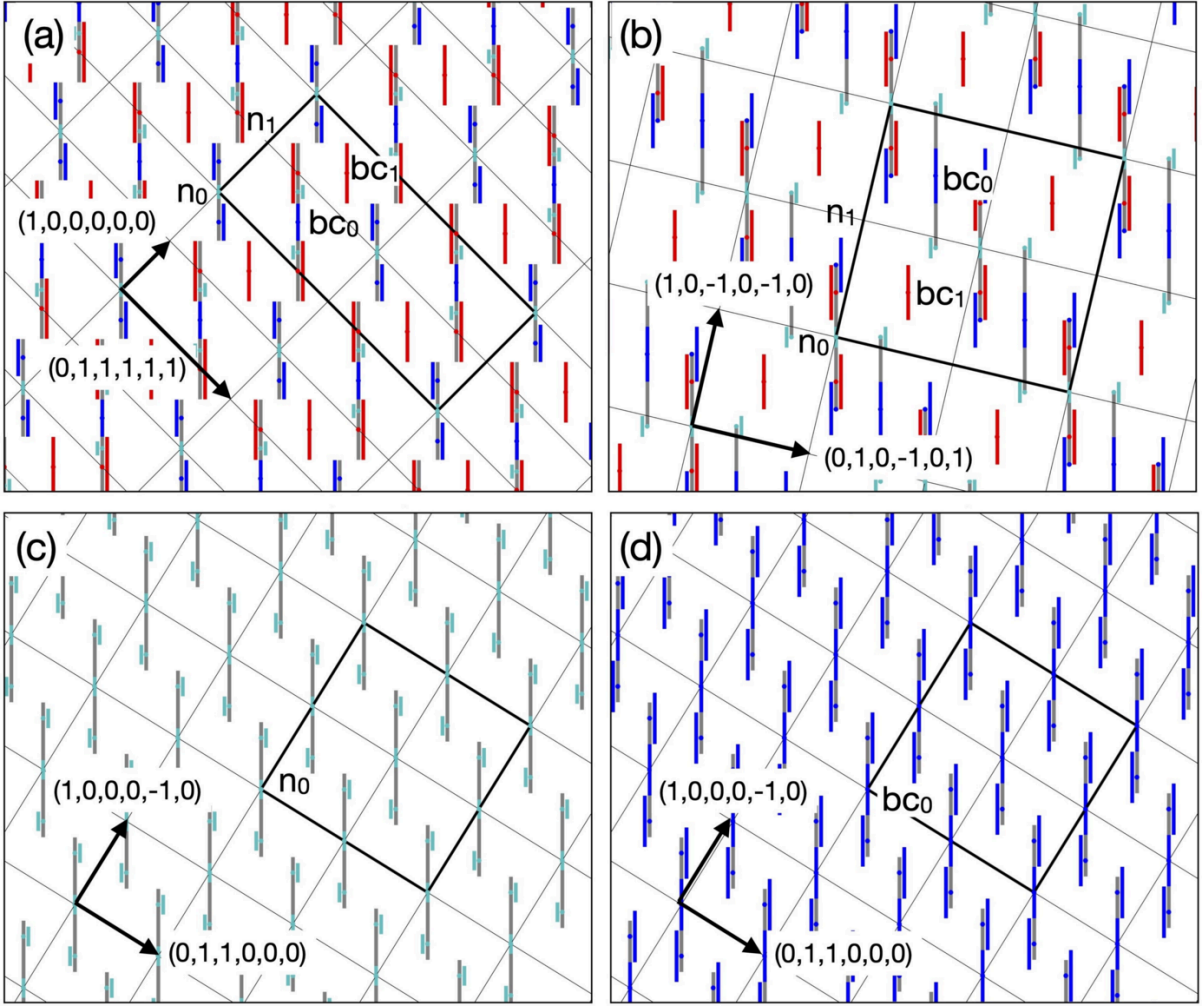
Arrangement of the shell-ODs on the 6D lattice. (*a*) Fivefold plane, (*b*) threefold plane, (*c*) twofold plane passing through n_0_, (*d*) twofold plane passing through bc_0_. Line segments shown in blue, cyan and red represent ODs for pM, pM′ and mB clusters, respectively. Line segments shown in grey represent the three ODs of the scaled-KG model. Some shell-ODs are slightly shifted along 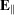 from their original positions to make it easier to see the size of each OD. The thick black line shows the 2D section of the 6D unit cell.

**Figure 12:**
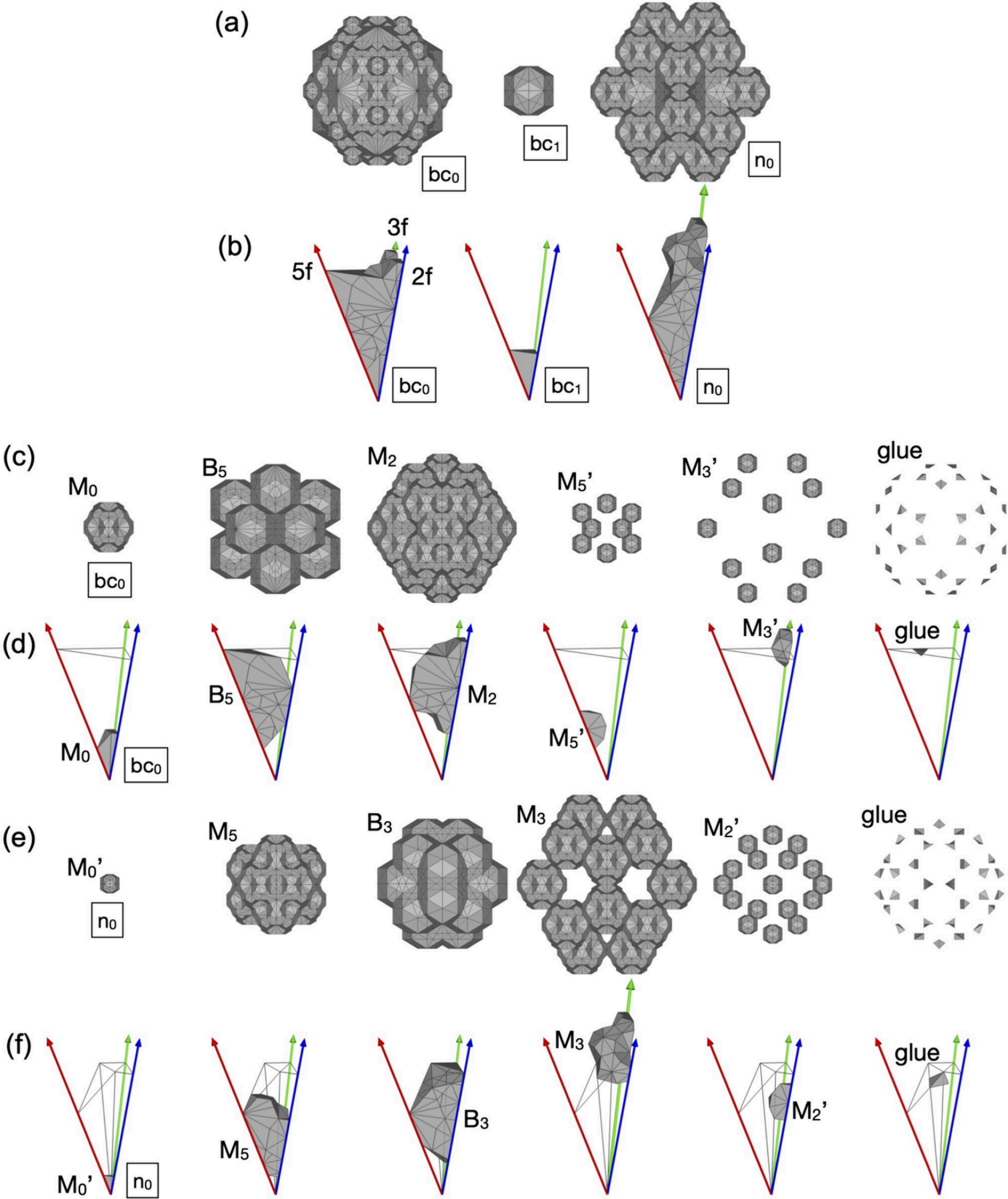
Shell-ODs for the pM+pM′ and mB clusters in the 6D model. (*a*) Overall shape of the shell-ODs at bc_0_, bc_1_ and n_0_. (*b*) The asymmetric units of the ODs in (*a*). (*c*) The shell-ODs at bc_0_ for M_0_, B_5_, M_2_, M_5_′ and M_3_′ shells. (*d*) The asymmetric units of the ODs in (*d*). (*e*) The shell-ODs at n_0_ for M_0_′, M_5_, B_3_, M_3_ and M_2_′ shells. (*f*) The asymmetric units of ODs in (*e*). Two ODs for the glue atoms, which are centred at bc_0_ and n_0_, are also drawn in the figure.

Fig. 11 ▸ shows the arrangement of the resulting shell-ODs on the 6D lattice. The shell-ODs are centred at bc_0_, bc_1_ and n_0_ positions. The overall ODs in the present model can be considered as an extension of the scaled-KG model with additional ODs outside of the 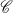 and 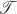. Those ODs describe partially occupied sites in pM+pM′ clusters. The 6D coordinates and volume of each shell-OD are summarized in Table 2 ▸, and the ODs projected onto the 3D 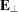 are presented in Fig. 12 ▸. As seen in Figs. 11 ▸ and 12 ▸, the shell-ODs for the M_2_ and M_3_′ shells centred at bc_0_ extend partially outside the 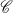 in the scaled-KG model. Similarly, the shell-OD for the M_3_ shell centred at n_0_ extends partially outside the 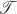. Since the scaled-KG model fulfils the closeness conditions, these shell-ODs generate unphysical short interatomic distances in the atomic structure, which will be discussed in Section 4.3.

The shell-ODs mentioned above represent approximately 99.46% of the three ODs of the scaled-KG model, namely 99.46% of the structure belongs to at least one of the pM+pM′ and mB clusters. The remaining 0.54%, which is obtained by removing the shell-ODs from the three ODs, corresponds to two ODs situated at bc_0_ and n_0_ sites, which are shown in Figs. 12 ▸(*d*) and 12 ▸(*f*), respectively, and the volume of the asymmetric unit is (89 − 55τ)/3 and (−55 + 34τ)/3. These ODs generate atomic sites called ‘glue’ atoms that do not belong to the clusters. The cluster covering ratio in the present model is much larger than that in the KG model, which is approximately 95% (Gratias *et al.*, 2000 ▸). Even the extended version of the KG model, which takes into account an extended Bergman cluster of 6-shells, only reaches approximately 97.73% (Duneau, 2000 ▸).

### 4.3. Subdivision of ODs

**Figure 13:**
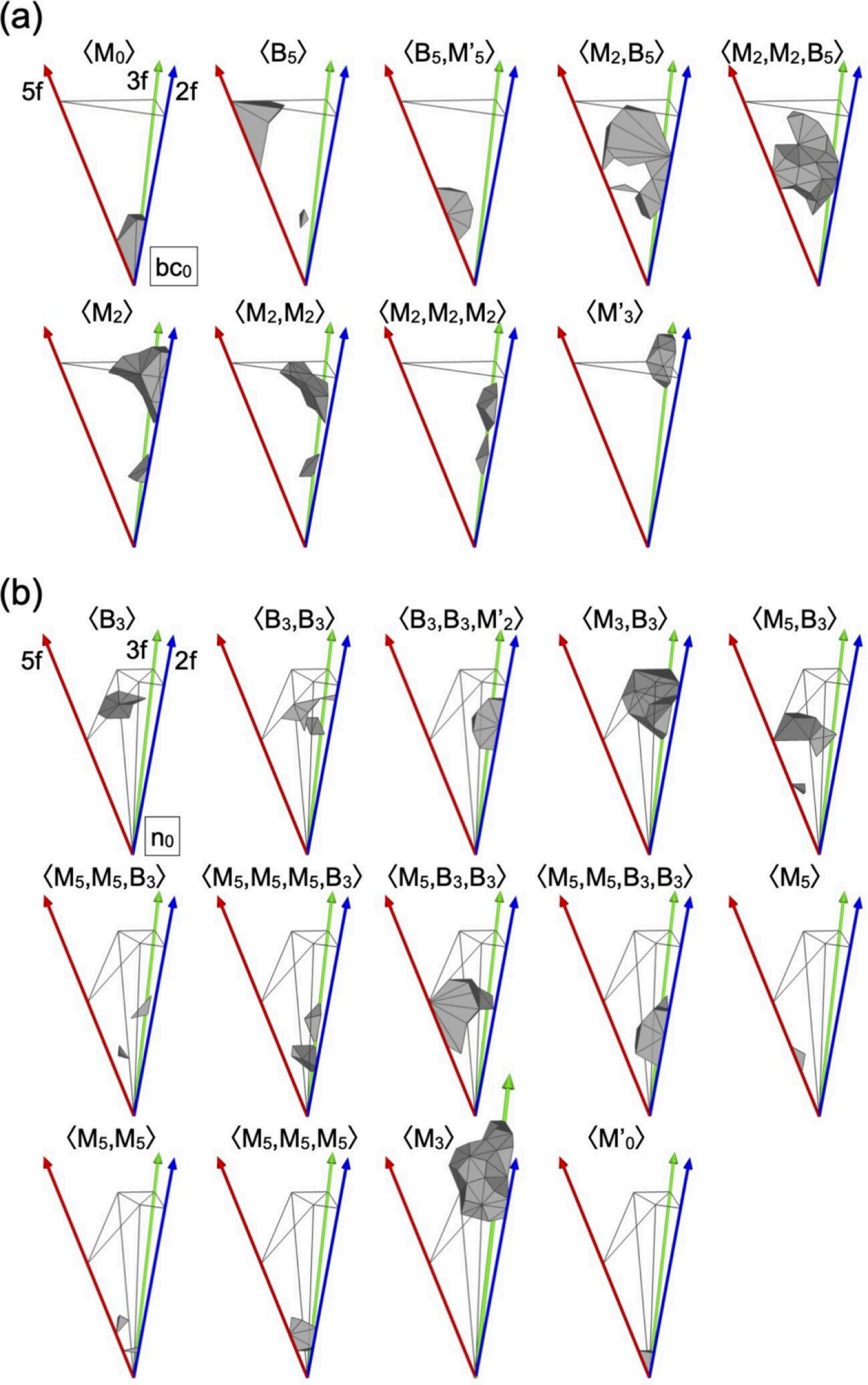
Subdivisions of the asymmetric unit of the ODs at (*a*) bc_0_ and (*b*) n_0_ according to the site classes. The frames in (*a*) and (*b*) drawn in grey indicate the 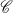 and 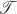 in the scaled-KG model, respectively.

As mentioned in Section 2, occupational and positional disorder in the Al–Pd–Cr–Fe 3/2 *c*-AC are well characterized by the site class (Fujita *et al.*, 2013 ▸); therefore, the knowledge of site class in the model of *F*-type *i*-QCs is of great importance. A site class in the *i*-QCs is determined by identifying shell-ODs that intersect each other when projected onto the 3D 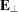. It turns out that there are in total 24 classes of atomic sites in the pM+pM′ and mB clusters. The ODs corresponding to the 24 classes (hereafter, referred to as class-ODs) are presented in Fig. 13 ▸, and the volume of the class-ODs and their frequency are summarized in Table 3 ▸.

As seen in Fig. 13 ▸, some class-ODs extend outside of the 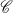 and 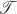 in the scaled-KG model. Because these ODs result in unrealistic short interatomic distances in the atomic structure, they must be distinguished from other class-ODs which are located inside of the 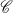 and 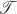. In the following, we subdivide the corresponding classes into several subclasses. Hereafter, we express the *j*th subclass of the 〈X^1^, X^2^, ..., X^*n*^〉 class by [X^1^, X^2^, ..., X^*n*^]^*j*^.

**Figure 14:**
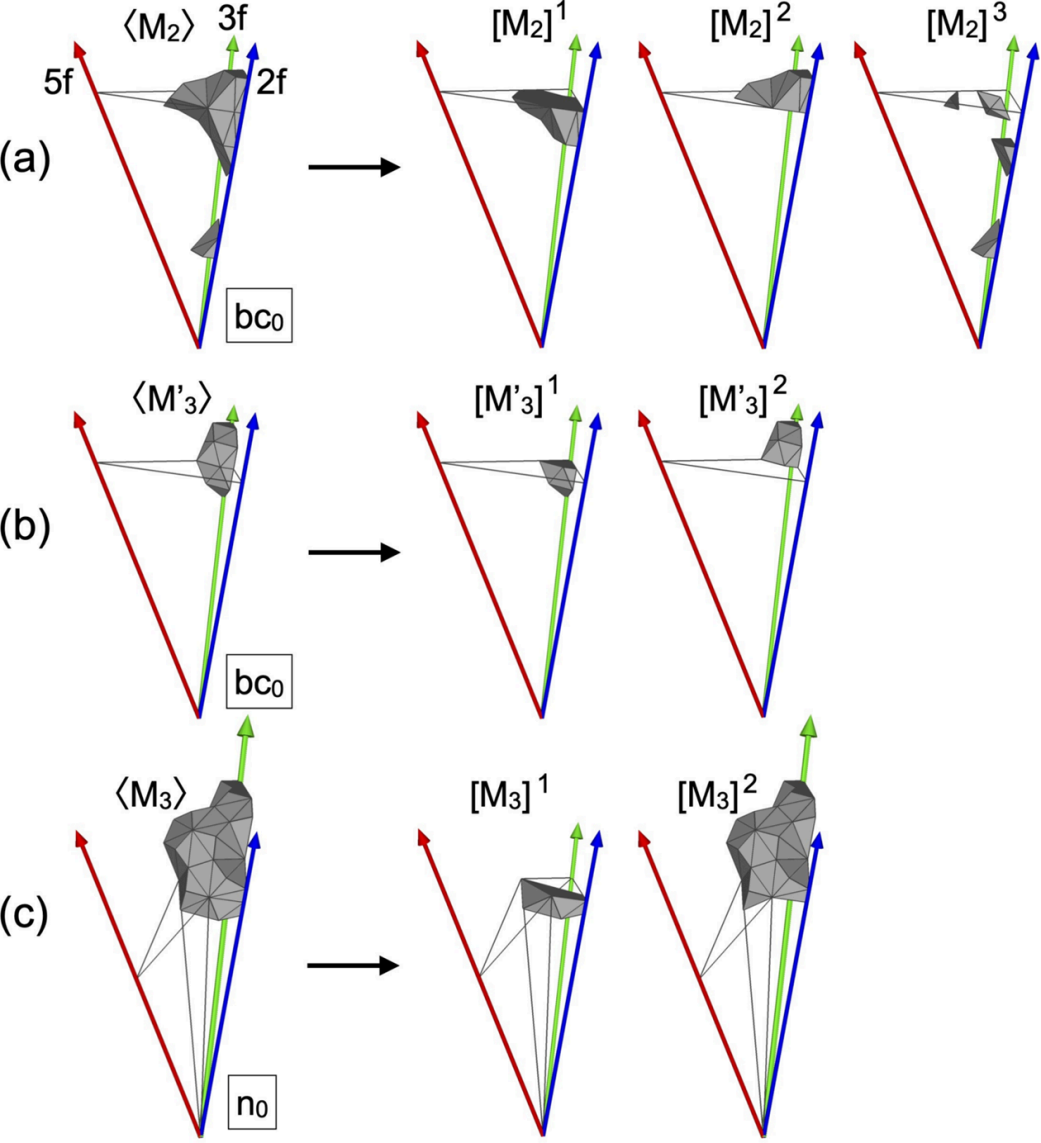
Class-ODs extending outside of the scaled-KG model. Subdivision of the class-ODs of (*a*) 〈M_2_〉 at bc_0_, (*b*) 〈M_3_′〉 at bc_0_ and (*c*) 〈M_3_〉 at n_0_. Only the asymmetric units are shown. Frames drawn in grey in (*a*), (*b*) indicate 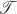 and that in (*c*) indicates 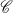 in the scaled-KG model.

The atomic site on the M_2_ shell belongs to one of the classes 〈M_2_〉, 〈M_2_, B_5_〉, 〈M_2_, M_2_〉, 〈M_2_, M_2_, B_5_〉 and 〈M_2_, M_2_, M_2_〉. As seen in Fig. 13 ▸(*a*), only the class-OD of the 〈M_2_〉 class partially extends outside of 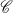, while those for the other classes are fully located inside of 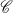. The 〈M_2_〉 class can be divided into three subclasses, [M_2_]^1^, [M_2_]^2^ and [M_2_]^3^, and the ODs corresponding to these subclasses (hereafter, referred to as subclass-ODs) are obtained as shown in Fig. 14 ▸(*a*). The subclass-ODs of [M_2_]^1^ and [M_2_]^2^ result in the generation of an unphysical short interatomic distance between two M_2_ sites situated on the *b*-linkage of two pM clusters, with a distance of approximately 1.8 Å for *a* = 2.8 Å [see Fig 2 ▸(*a*)]. The subclass-ODs of [M_2_]^1^ and [M_2_]^2^ have a mirror-symmetric form with respect to each other, with the volume given by (−637 + 394τ)/6 in the asymmetric unit, and the latter is extending outside of 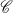. A pair of two sites generated by these subclass-ODs is interpreted as splitting sites with an occupation probability equal to 1/2, as for those in the 3/2 *c*-AC. The atomic sites generated by the subclass-OD of [M_2_]^3^, whose volume in the asymmetric unit is given by (335 − 207τ)/3, are not related to the splitting sites.

The edge length of M_3_ and M′_3_ shells, which is approximately 1.8 Å for *a* = 2.8 Å, is too short to occupy atoms at the 20 vertices of the shell at the same time. Therefore, the occupation probability at these sites should be less than unity, as for those observed in the 3/2 *c*-AC. We consider the occupational disorder on the M_3_ and M′_3_ shells as follows.

The atomic sites in the M_3_′ shell belong to the 〈M_3_′〉 class. The corresponding class-OD partially extends outside of 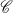 [see Fig. 13 ▸(*a*)]. Consequently, the class is subdivided into two subclasses, [M_3_′]^1^ and [M_3_′]^2^, and the corresponding subclass-ODs are obtained as shown in Fig. 14 ▸(*b*). The volume in the asymmetric unit of the former and the latter is given as (−385 + 238τ)/6 and (−715 + 442τ)/6, respectively. The number of atoms in the M_3_′ shell is 7, provided that the atomic sites of [M_3_′]^1^ are fully occupied by atoms and those of [M_3_′]^2^ are vacant. In this case, no short interatomic distance less than approximately 2.9 Å for *a* = 2.8 Å appears on the M_3_′ shell.

As mentioned above, an atomic site on the M_3_ shell belongs to the site class either 〈M_3_, B_3_〉 or 〈M_3_〉. The former distinguishes the atomic site that is shared by the M_3_ and B_3_ shells on the *c*-linkage, while the latter represents the rest of the M_3_ site. The class-OD for the 〈M_3_, B_3_〉 class fully intersects with 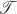, while that for the 〈M_3_〉 partially extends outside of 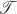 [see Fig. 14 ▸(*c*)]. Consequently, the 〈M_3_〉 class is subdivided into two subclasses, [M_3_]^1^ and [M_3_]^2^. The subclass-ODs of [M_3_]^1^ and [M_3_]^2^ are obtained as shown in Fig. 14 ▸(*c*). The former fully intersects with 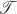, while the latter is located outside of it. The volume in the asymmetric unit of the subclass-ODs is given as (149 − 92τ)/6 and (−1033 + 640τ)/6 for [M_3_]^1^ and [M_3_]^2^, respectively. If we assume the sites generated by 〈M_3_, B_3_〉 are fully occupied and those by 〈M_3_〉 are vacant, as in the Al–Pd–Cr-Fe 3/2 *c*-AC (Fujita *et al.*, 2013 ▸), the average number of atoms in the M_3_ shell is approximately 6.14. Here, the number of atoms in each shell depends on the local cluster configurations, and ranges between 4 and 7, because the number of *c*-linkages in the local configuration of pM clusters ranges from 4 to 7 (see Table 1 ▸).

### 4.4. Point density

The point density of a structure model is a fundamental quantity. In the case of *i*-QCs, it is given by ρ = Ω_⊥_/*V*_cell_, where 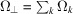 is the summed volume of ODs Ω_*k*_ in the 3D 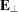 and *V*_cell_ = det|*M*| is the unit-cell volume of the 6D icosahedral lattice. Here, *M* = [*Q*, *Q*′] is a 6 × 6 matrix given by *Q* and *Q*′ in Appendix *A*[App appa]. The point density of the scaled-KG model is given by (κ*a*)^3^ρ = (−67 + 44τ)/20 ≃ 0.2097, where κ = 1/(τ + 2)^1/2^.

The point density of our 6D model depends on how many atoms are present in each M_3_ and M′_3_ shell, which is expressed by a common parameter *x* as follows. For the sake of simplicity, we consider the following conditions: (i) the class-OD for the 〈M_3_, B_3_〉 class is fully occupied; (ii) the occupational probability of the subclass-ODs [M_2_]^1^ and [M_2_]^2^ is equal to 1/2, as considered in Section 4.3. To derive the point density, the occupational probability has to be set individually for each local configuration of the pM cluster; therefore, the subclass-ODs [M_3_]^1^ and [M_3_]^2^ are subdivided in terms of the local configuration. The resulting volume of the subdivided subclass-ODs for the [M_3_]^1^ and [M_3_]^2^ subclasses is listed in Table 4 ▸. Considering all the above conditions, the point density is given by 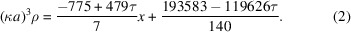
This formula is validated only for 7 ≤ *x* ≤ 20, since the number of atoms that belong to the site class [M_3_, B_3_] inside each pM cluster is in the range 4 to 7 owing to condition (1).

The occupational disorder inside pM clusters has been investigated by several research groups. In a structure model for the Al–Pd–Mn *i*-QC obtained based on *ab initio* simulation, a central Mn atom at the pM cluster is surrounded by eight Al atoms (Quandt & Elser, 2000 ▸). A more energetically favourable structure model was obtained, in which the central Mn atoms are surrounded by nine instead of eight Al atoms (Zijlstra *et al.*, 2005 ▸). In addition, a recent study of the Al–Cu–Fe *i*-QC showed the presence of nine to ten Al atoms around the central Fe atom in the pM cluster (Mihalkovič & Widom, 2020 ▸). From equation (2) with *x* = 9, one obtains (κ*a*)^3^ρ ≃ 0.221, which is slightly larger than that of the KG model. To discuss further occupational disorder of the pM cluster, a reliable density measurement of *F*-type Al-based *i*-QCs is needed, and this is a subject for future research.

### 4.5. Real-space structure and reorganization of cluster arrangement

**Figure 15:**
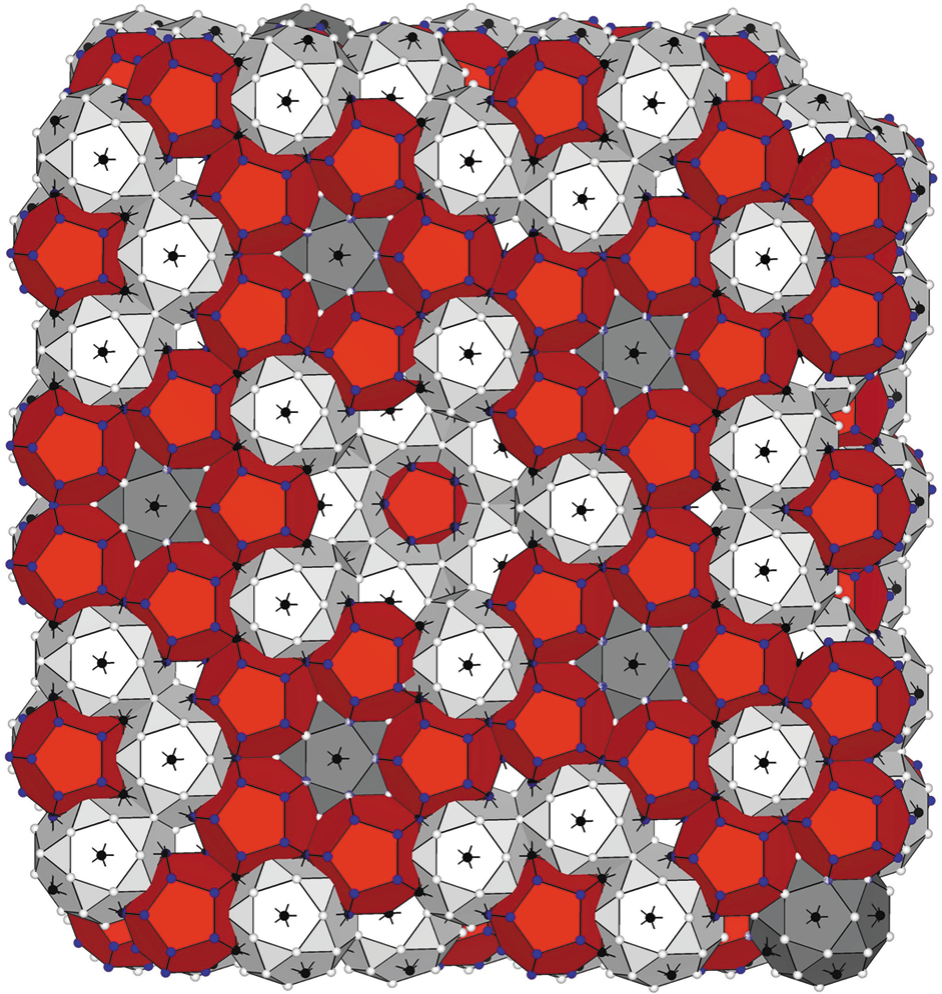
Cluster arrangement in **E**_∥_. The M_2_, M_2_′ and B_3_ shells are drawn in white, grey and red, respectively.

Fig. 15 ▸ shows a slab structure derived from the present 6D model. The real-space structure exhibits one-to-one correspondence of each atomic position to the site class. As shown above, the occupational and positional disorder are characteristic of the pM clusters present in the specific site classes, so that the present model can allow us to handle precisely the disorder in a structure refinement using diffraction intensities, which is a research topic for the future.

Although the present 6D model is a deterministic one, it is important to recognize that the higher-dimensional approach incorporates arbitrariness. As pointed out by Elser (1996 ▸), the degrees of freedom in the real-space structure must be specified in detail to go beyond the higher-dimensional approach. In the following, we consider possible local reorganization of pM+pM′ and mB clusters in the real-space structure.

**Figure 16:**
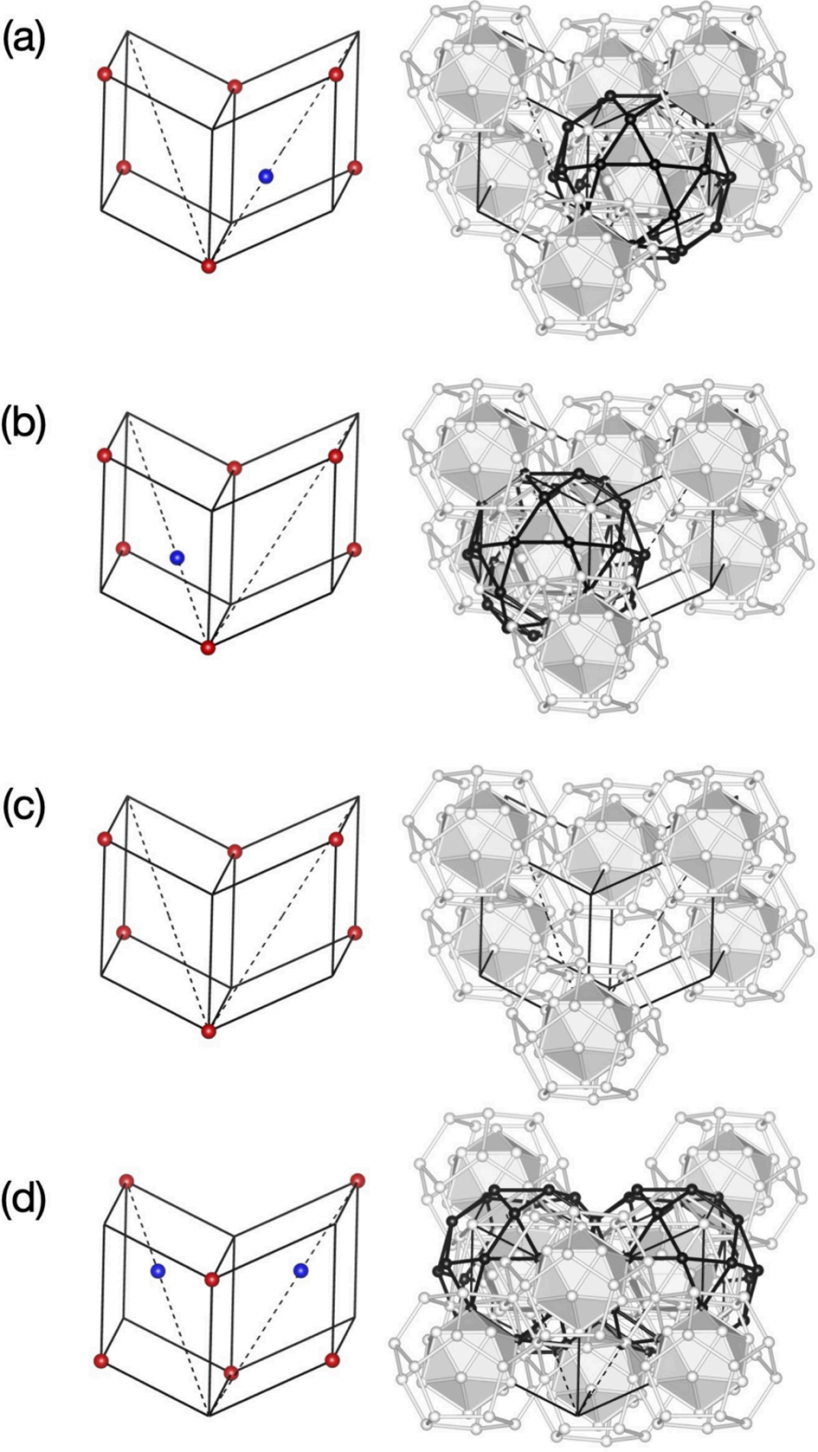
Decoration of two adjacent acute rhombohedra with edge length of τ^2^*a* by pM and mB clusters. (*a*), (*b*) The case where one of the two acute rhombohedra contains a pM cluster. (*c*) The case where acute rhombohedra contain no pM cluster. (*d*) The case where each acute rhombohedron includes a pM cluster. (Left) Cluster centre positions. The blue and red balls represent the centres of the pM and mB clusters, respectively. (Right) Arrangement of the pM and mB clusters. Only the cluster shells on the unit are drawn for clarity.

As mentioned above, the arrangement of pM+pM′ and mB clusters in the present model can be understood as a decoration of the 3D AKN tiling with edge length of τ^2^*a*. The pM′ and mB clusters are located at specific positions: the former is located at all ‘even’ 12-fold vertices, and the latter is situated at all ‘odd’ vertices of the tiling. On the other hand, the pM clusters may or may not occupy the positions where the long diagonal of an acute rhombohedron is divided τ:1 from the ‘even’ vertex towards the ‘odd’ vertex (see Fig. 16 ▸). This arrangement, which tolerates some arbitrariness in the choice of pM cluster centre to be eliminated from a pair of two possible positions, avoids the short distance of (τ − 1)*b*/2 between two neighbouring pM clusters. Therefore, the arrangement of pM clusters exhibits some degrees of freedom in the real-space structure.

Focusing on a unit consisting of two adjacent acute rhombohedra in the structure, we notice the possibility of local reorganization of a pM cluster in the unit. When the two acute rhombohedra share their ‘odd’ vertices on the threefold axes, three different arrangements of the pM cluster are possible [see Figs. 16 ▸(*a*), 16 ▸(*b*), 16 ▸(*c*)]. Interestingly, when a pM cluster is situated at the position on the body-diagonal in one acute rhombohedron, the atomic sites related to the possible cluster centre position on another acute rhombohedron form almost perfect shells of a pM cluster. Therefore, a reorganization of the pM cluster arrangement may proceed via atomic diffusion involving a few atoms. When the two acute rhombohedra share their ‘even’ vertices, two pM clusters can be arranged inside the unit, as shown in Fig. 16 ▸(*d*).

**Figure 17:**
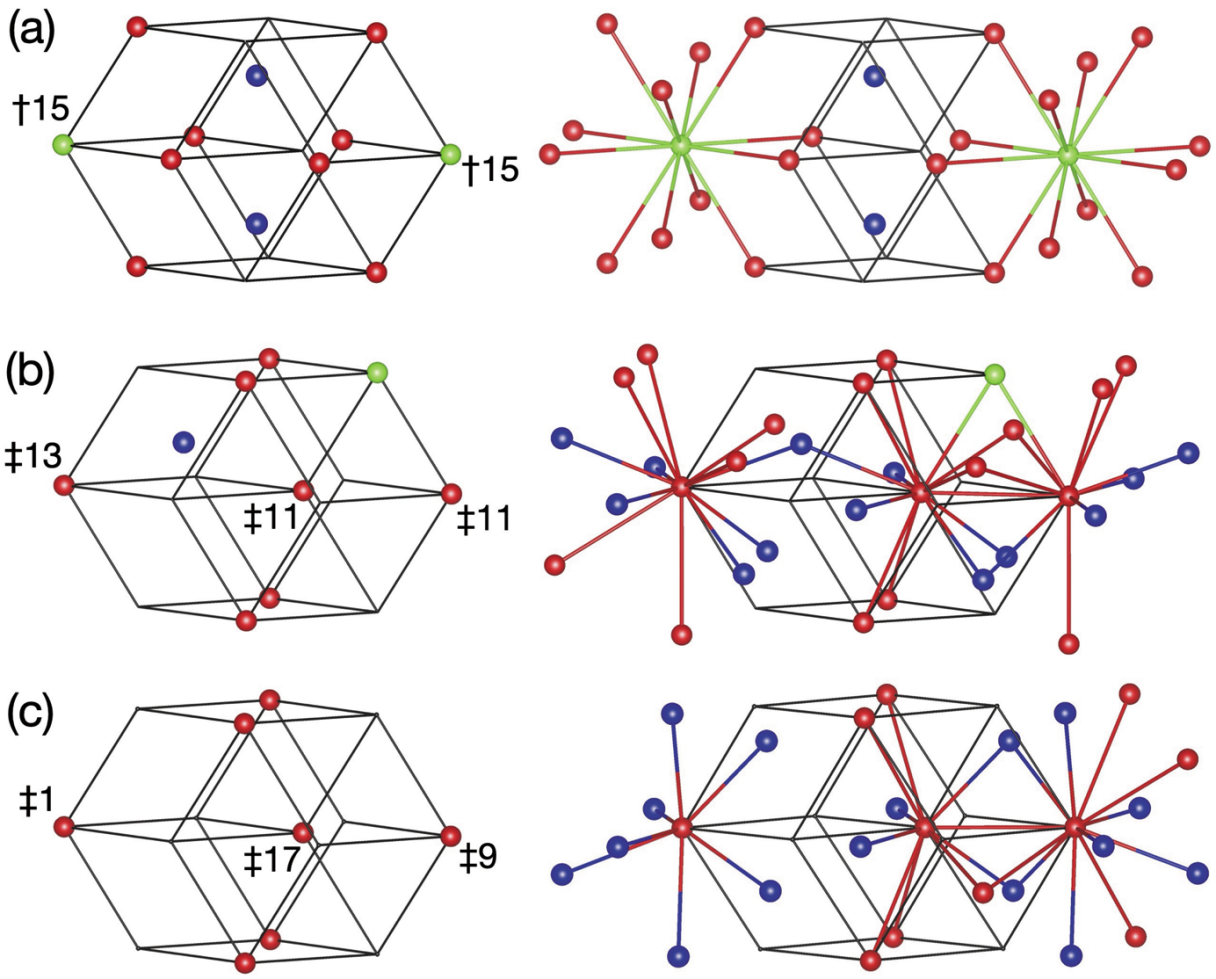
Three examples of rhombic dodecahedra found in the real-space structure derived from the 6D model. Each rhombic dodecahedron is composed of two acute rhombohedra and two obtuse rhombohedra. Units exhibit (*a*) two pM clusters, (*b*) one pM cluster and (*c*) no pM cluster inside. (Left) Cluster centre positions. The blue, green and red balls present the cluster centre positions of the pM, pM′ and mB clusters, respectively. The local environments for a few clusters are indicated. (Right) Cluster linkages and cluster centre positions of the first neighbouring clusters are described.

Another interesting, simple unit worth considering is the rhombic dodecahedron, which consists of two acute and two obtuse rhombohedra. This unit can be obtained with two different rhombohedral arrangements that do not change its external shape. The relationship between these two configurations is considered as a tile flip (or phason flip) which results in the vertex located inside the unit changing its position. Fig. 17 ▸ shows three examples of the arrangement of pM+pM′ and mB clusters in the rhombic dodecahedron units found in the real-space structure. In Fig. 17 ▸(*a*), two pM clusters are situated inside the rhombic dodecahedron, while no mB cluster exists. In Figs. 17 ▸(*b*) and 17 ▸(*c*), the arrangement of mB clusters differs from that in the case of Fig. 17 ▸(*a*), and the number of pM clusters is one and zero, respectively. Furthermore, when the unit is organized as in Figs. 17 ▸(*b*) and 17 ▸(*c*), the tile flip of the central vertex in the rhombic dodecahedron leads to the reorganization of the mB cluster. An mB cluster situated at one side of the central vertex, and the atomic sites around another side of that form almost perfect shells of the mB cluster; therefore, the reorganization of the mB clusters may proceed via atomic diffusion involving a small number of atoms.

**Figure 18:**
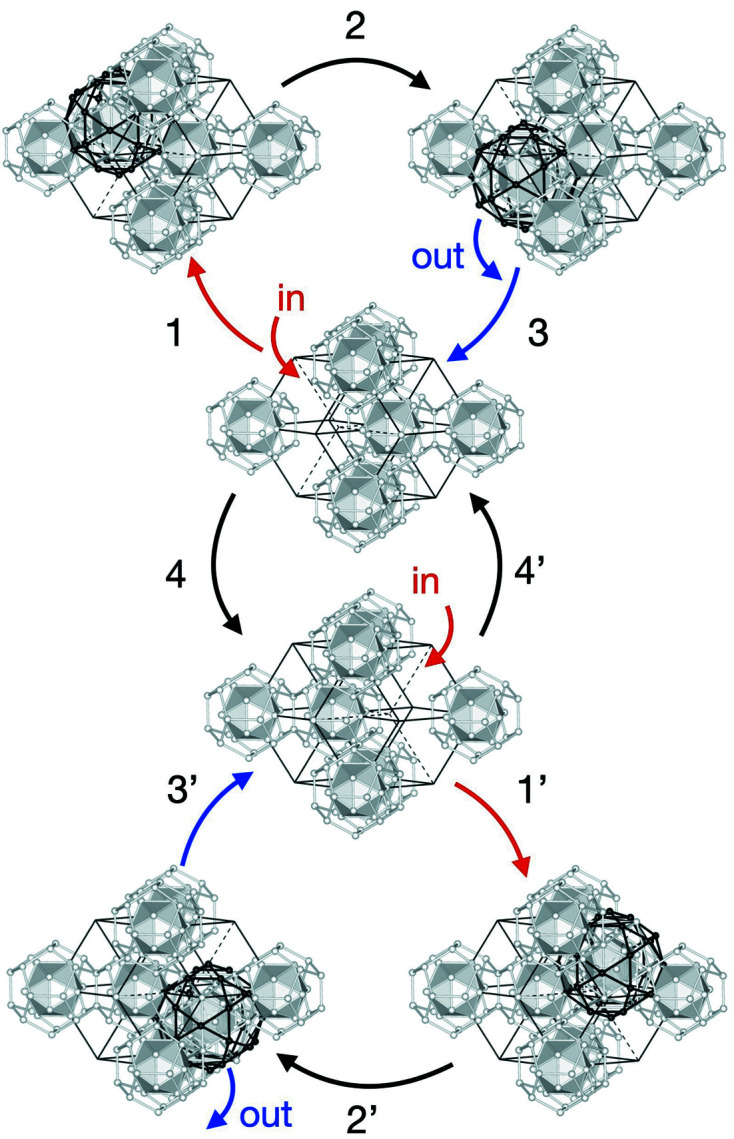
A cyclic reorganization of the pM and mB clusters in a rhombic dodecahedron unit. Only the cluster shells on the unit are drawn for clarity.

On account of the above, a cyclical local reorganization of pM and mB clusters is possible in various manners in rhombic dodecahedron units. An example of reorganization of the clusters is shown in Fig. 18 ▸. When we start with a unit corresponding to that shown in Fig. 17 ▸(*c*), the reorganization can occur in the following steps: (i) a pM cluster moves in the rhombic dodecahedron from an acute rhombohedron neighbouring to that; (ii) the pM cluster moves to another acute rhombohedron in the rhombic dodecahedron; (iii) the pM cluster goes out to a neighbouring acute rhombohedron; (iv) a tile flip accompanies reorganization of the mB cluster at the central vertex. The latter follows a similar reorganization by steps (i′) to (iv′) as described in the figure.

The local reorganization of the pM and mB clusters represents phason flips that correspond to the local distortion of the 3D section. Correlated phason flips lead to long-wavelength phason fluctuations (phason modes) that are expressed in the framework of the hydrodynamic theory of QCs (Kalugin *et al.*, 1985*a* ▸,*b* ▸; Bak, 1985*a* ▸,*b* ▸; Lubensky *et al.*, 1985 ▸). The detail of the phason fluctuations has been experimentally investigated in the Al–Pd–Mn *i*-QC (de Boissieu *et al.*, 1995 ▸, 2007 ▸; Boudard *et al.*, 1996 ▸; Letoublon *et al.*, 2001 ▸; Francoual *et al.*, 2003 ▸), and a summary of the results may be found elsewhere (de Boissieu, 2008 ▸). The root-mean-square deviation, 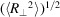, of the phason fluctuations in the Al–Pd–Mn *i*-QC was estimated to be approximately 1.4 Å (de Boissieu *et al.*, 1994*b* ▸). The radius of 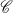 is τ*a* (≃ 4.5 Å), τ[(9 − 5τ)/5]^1/2^*a* (≃ 4.1 Å) and τ[(2 + τ)/5]^1/2^*a* (≃3.9 Å) along the fivefold, threefold and twofold directions, respectively; it means that the 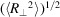 is roughly 30% of these OD radii or more. This implies that local reorganization of the clusters may occur frequently.

Since the diffraction intensities provide information on a spatial and temporal averaged structure, the local reorganization of clusters results in additional sites with fractional occupancies. These atomic positions can be generated by adding corresponding ODs. For instance, one can model the pM clusters after the reorganization with the OD shown in Fig. 8 ▸(*c*). In this case, a proper subdivision of the occupation domains is necessary to determine the occupancy of the atomic sites, because the two pM clusters separated by a short distance (τ − 1)*b*/2 will heavily interpenetrate each other and share some atomic sites. Construction of such a 6D model is a subject for future research.

## 5. Comparison with other models

### 5.1. Model by Elser

Elser (1996 ▸) developed a random tiling model and proposed an atomic ‘escapement mechanism’ that implements tile flips. The model structure mainly consists of two kinds of cluster named ‘Bergman dodecahedron’ and ‘Mackay volume’, each of which is similar to mB and pM clusters in our model, respectively. The former is located on all ‘odd’ vertices of an inflated 3D AKN tiling with an edge length of τ^2^*a*. In this model, two Bergman dodecahedra are connected to each other by a twofold *b*-linkage, which corresponds to that shown in Fig. 2 ▸(*a*), along a shorter diagonal of the rhombus face of each rhombohedron. The gap space of the edge-sharing network of the Bergman dodecahedra is filled with the Mackay volume located on every ‘even’ vertex and a small interstitial volume found on the threefold axis of each acute rhombohedron. Furthermore, the Bergman dodecahedron and the Mackay volume, which are situated at the vertices of an edge, are connected by the τ^2^*a*-linkage, similar to that shown in Fig. 7 ▸. In addition, the *b*-linkage of the Mackay volumes, which corresponds to that shown in Fig. 2 ▸(*b*), is found along the shorter diagonal of the rhombus face of each rhombohedron.

Like Elser’s model, the mB cluster in our model is located at all the ‘odd’ vertices of the acute and obtuse rhombohedra with the same size, as described in Section 4.1. On the other hand, the position of pM+pM′ differs from that of the Mackay volume in Elser’s model: the pM+pM′ cluster is not located on all the ‘even’ vertices. Instead, the pM cluster is situated on a position that divides the longer body-diagonal of the acute rhombohedron by τ:1 from ‘even’ to ‘odd’ vertices [see Figs. 9 ▸(*a*) and 9 ▸(*b*)], and the pM′ cluster is located at all the ‘even’ 12-fold vertices of the tiling.

Furthermore, Elser’s model exhibits a short linkage with a distance of τ^−1^*c*, which is absent in our model, along the threefold axis of each obtuse rhombohedron. With this linkage, a Bergman dodecahedron and a Mackay volume heavily interpenetrate each other as follows: (i) a B_3_ site on the linkage is located at the central M_0_ site; (ii) six B_3_ sites are situated at the M_2_ shell; (iii) three B_5_ sites are situated at the M_5_ shell. In addition to these, three B_3_ sites are located close to the edge centre of the M_3_ shell. Consequently, the M_3_ shell shows an occupational disorder which depends on the configuration of neighbouring Bergman dodecahedra. This feature is similar to our model as described in Section 4.3.

### 5.2. Model by Gratias *et al.*

Gratias *et al.* (2000 ▸) presented a subdivision of the three ODs (see Fig. 5 ▸) originally proposed by Katz & Gratias (1993 ▸). The resulting atomic structure consists of two kinds of clusters, named Mackay’s (M) and Bergman’s (B) clusters, each of which is similar to the pM and mB clusters considered in the present study. In their model, the ODs for the cluster centres were derived as follows: (i) the M and M′ clusters are generated by two identical ODs at bc_0_ and n_0_, respectively, each of which is a 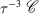; (ii) the B cluster is generated by the 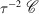 at bc_1_ (note that we mention the scaled-KG model here for comparison with our model). The former differs from both the OD of M_0_ and that of M_0_′ in the present model. The total volume of the asymmetric unit of the ODs for the centres of M and M′ clusters equals (13 − 8τ)/3, whereas that of the pM+pM′ clusters in the present study equals 13τ − 21; therefore, the distribution of the M and M′ clusters and their local configurations are essentially different from those in the present model. In fact, the number of M and M′ clusters is approximately 0.54 times that of the pM+pM′ clusters in the present model. In addition, unlike our model, the twofold *b*-linkage which links two M clusters is absent in the resulting atomic structure. On the other hand, the second is identical to the OD for B_0_ in our model, indicating that the distributions of B and mB clusters are the same.

The analysis of the cluster arrangement in Section 4 gives a new insight into the atomic arrangement in the KG model. The cluster centres are arranged at the vertices of the τ^2^-inflated AKN tiling and one of the two body-diagonal positions of acute rhombohedra dividing the diagonal to 1:τ. The body-diagonal position is sometimes empty, and so on. These rules apply in exactly the same way to the atom positions in the KG model, since the ODs for the atom positions of the scaled-KG model are related by the similarity transformation of order 2 to those of the cluster centres in the present model after shifting the origin to bc_1_.

We consider the AKN tiling with edge length of *a* instead of τ^2^*a* (note that *a* ≃ 2.8 Å). After the origin shift, the 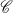 and 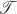 are at n_1_ and bc_0_ in the new coordinate system and form the odd vertices and body-diagonal positions of the AKN tiling with edge length of *a* as atom positions. On the other hand, the 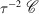 at n_0_ forms the ‘even’ 12-fold vertices (see Fig. 9 ▸). As a result, all odd vertices are generated, but only ‘even’ 12-fold vertices are created, while the 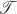 at bc_0_ partially generates the body-diagonal position in the acute rhombohedra.

### 5.3. Al–Pd–Mn *i*-QC model by Yamamoto *et al.*

Yamamoto *et al.* (2003 ▸) proposed a 6D structure model of Al–Pd–Mn *i*-QC based on the 2/1 *c*-ACs (Sugiyama *et al.*, 1998 ▸, 2002 ▸). In this model, five types of atomic clusters are situated at vertices and edge-centred positions of an inflated 3D AKN tiling with an edge length of 20 Å, and two body-diagonal positions of each acute rhombohedron. Because the cluster centre ODs in this model are 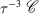 and 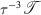, the arrangement of the clusters differs from that in the present model and is rather similar to the cluster arrangement in the KG model mentioned above.

### 5.4. Al–Cu–Fe *i*-QC by molecular dynamics simulation

Recently, Mihalkovič & Widom (2020 ▸) succeeded in realizing atomic arrangements of an *F*-type *i*-QC in the Al–Cu–Fe system, by molecular dynamics simulations using empirical oscillating pair potentials, and the simulation revealed the full dynamic evolution, including correlation among positions of partial and mixed occupation that represent phason fluctuations.

The resulting averaged atomic arrangement was interpreted by a packing of three types of atomic clusters: a small icosahedron (I), a pseudo-Mackay icosahedron (pMI) and a large pseudo-Mackay icosahedron (τ-pMI). The I cluster is composed of a central Cu atom surrounded by an icosahedron of Al_12−*x*_Cu_*x*_. The pMI cluster is composed of an inner Al_12−*x*_Fe surrounded by a large icosahedron and a large icosidodecahedron. The τ-pMI cluster is composed of similar shells with the pMI cluster and a surrounding icosahedral Al_60_Cu_12_(Fe,Cu)_30_ shell.

Although the atomic clusters that were used for the interpretation of the atomic structure differ from those in our model, we find the following correspondences. First, the I cluster corresponds to the first and second shells in the mB cluster. Second, the pMI cluster corresponds to the pM cluster. Third, the inner shells of the τ-pMI cluster correspond to the pM′ cluster. Last, the outer fourth shell of the pMI cluster is composed of atoms in the third shells of mB clusters.

Furthermore, it was found that the distribution of the averaged simulated atomic positions in 3D 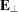, which were obtained by lifting the atoms into the 6D space, fit the three ODs in the KG model. The authors named these AS_1_, AS_2_ and B_1_, each of which corresponds to 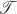, 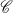 and 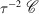, respectively, in the scaled-KG model (Fig. 5 ▸). Interestingly, the occupation probability in the outer part of the distribution was found to be lower and the distribution slightly extended outside of the ODs in the KG model. This finding is associated with the class-ODs for 〈M_3_〉, 〈M_2_〉 and 〈M_3_′〉 in the present model, and supposed to be related to the positional and occupational disorder. It is also interesting to note that the elemental distribution on AS_2_ is clearly distinguished, and the distribution form of Cu atoms corresponds well to the 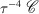 OD for the M_5_′ shell in the present model. These facts indicate that considering pM′ clusters is necessary to analyse the selective atomic occupations in real *i*-QCs.

## 6. Summary and concluding remarks

A 6D model of the *F*-type *i*-QCs consisting of pM and mB clusters was derived. The model is properly described by the coordinate system with τ^−1^ times the lattice constant (*a* ≃ 2.8 Å). Overall ODs for all atoms except for those corresponding to disordered sites are the same as those in the scaled-KG model. They are subdivided based on the archetype ODs which generate the centres of the pM and mB clusters. The archetype ODs have similar shapes to the ODs in the scaled-KG model, but their size is deflated by τ^−2^. The exact shape of the ODs for different local environments of the pM and mB clusters and the frequency of each cluster configuration are given. The model results in a ratio of the pM and mB clusters equal to approximately 46.7% and 53.3%, respectively, close to those observed in the structure model of the 3/2 *c*-AC. The shell-ODs for the pM and mB clusters cover approximately 99.46% of the ODs in the scaled-KG model, and the remaining 0.54% of the ODs describe the glue-atom sites. Furthermore, the shell-ODs are decomposed into smaller ODs according to site class. The exact volume of each OD is given together with the frequency of each class. The shell-ODs for the M_3_, M_2_ and M_3_′ shells, which extend outside of the ODs in the scaled-KG model, were subdivided into smaller ODs. The subdivision of the ODs based on the site class is definitely crucial in a structure refinement to deal with the occupational and positional disorder that are characteristic of the *F*-type Al-based *i*-QCs.

## Figures and Tables

**Table 1:** Local configurations of pM+pM′ and mB clusters The serial numbers in the first column represent each local configuration. The characters († and ‡) distinguish the pM+pM′ and mB clusters, respectively. The coordination number (CN) of the first neighbouring clusters is given in the second column. The third, fourth and fifth columns correspond to the number of constituting linkages Z_*a*_, Z_*b*_, Z_*c*_ for τ^2^*a*, *b*- and *c*-linkages, respectively. The volume of the OD in the asymmetric unit and frequency (in percentage) for each local configuration are given in the seventh and eighth columns, respectively.

No.	CN	*Z*_*a*_	*Z*_*b*_	*Z*_*c*_	6D vector	Volume	Frequency
pM cluster							
†1	7	0	0	7	(2, 0, 0, 0, − 1, 0)	(233 − 144τ)/6	0.701488…
†2	8	0	1	7	(3, 1, 1, 0, − 2, 0)	(−987 + 610τ)/6	0.165599…
†3	9	0	2	7	(4, 1, 0, 0, − 1, 1)	(1597 − 987τ)/3	0.204691…
†4	10	0	3	7	(5, 0, 0, 1, 0, 1)	(−987 + 610τ)/3	0.331198…
†5	10	0	3	7	(3, 1, − 1, − 1, − 2, 1)	(−987 + 610τ)/3	0.331198…
†6	12	0	5	7	(3, − 1, − 1, 0, − 2, 0)	(68 − 42τ)/3	19.232273…
†7	11	0	4	7	(4, 0, − 1, 0, − 1, 1)	(610 − 377τ)/3	0.535889…
†8	11	0	5	6	(3, 0, 0, 0, − 3, 0)	(−55 + 34τ)/6	2.971550…
†9	12	0	6	6	(3, − 1, − 1, 0, − 2, − 1)	(−199 + 123τ)/3	8.213161…
†10	10	0	6	4	(3, 0, − 1, 0, − 2, 0)	(322 − 199τ)/3	5.076013…
†11	11	0	6	5	(3, 0, 0, 0, − 3, − 1)	(−521 + 322τ)/3	3.137148…
†12	11	0	5	6	(4, 0, − 1, 0, − 1, 0)	(233 − 144τ)/3	1.402975…
†13	9	0	5	4	(2, 0, − 2, − 1, − 2, 1)	(−377 + 233τ)/3	0.867086…
†14	10	0	5	5	(2, 0, − 1, − 1, − 3, 0)	(610 − 377τ)/3	0.535889…
pM′ cluster							
†15	12	12	0	0	(0, 0, 0, 0, 0, 0)	(−55 + 34τ)/6	2.971550…
mB cluster							
‡1	7	0	0	7	(2, 0, 0, 0, − 1, 0)	(233 − 144τ)/6	0.701488…
‡2	8	0	1	7	(3, 1, 1, 0, − 2, 0)	(−987 + 610τ)6	0.165599…
‡3	9	0	2	7	(4, 1, 0, 0, − 1, 1)	(1597 − 987τ)/3	0.204691…
‡4	11	1	5	5	(13, 1, 1, 1, 1, 1)/2	(−987 + 610τ)/6	0.165599…
‡5	10	0	3	7	(3, 1, − 1, − 1, − 2, 1)	(−987 + 610τ)/3	0.331198…
‡6	10	0	3	7	(5, 0, 0, 1, 0, 1)	(−2584 + 1597τ)/3	0.126506…
‡7	10	0	4	6	(5, 0, 0, 1, 0, 0)	(4181 − 2584τ)/6	0.039093…
‡8	12	0	5	7	(3, − 1, − 1, 0, − 2, 0)	(610 − 377τ)/3	0.535889…
‡9	11	0	4	7	(4, 0, − 1, 0, − 1, 1)	(1597 − 987τ)/3	0.204691…
‡10	11	0	5	6	(4, 0, − 1, 0, − 1, 0)	(−987 + 610τ)/6	0.165599…
‡11	12	1	6	5	(11, 1, − 1, − 1, − 1, 1)/2	(−987 + 610τ)/6	0.165599…
‡12	13	1	7	5	(9, − 1, − 1, − 1, − 3, − 1)/2	(657 − 406τ)/3	35.327397…
‡13	12	0	6	6	(3, − 1, − 1, 0, − 2, − 1)	(−1364 + 843τ)/3	1.198284…
‡14	11	0	6	5	(3, 0, 0, 0, − 3, − 1)	(−288 + 178τ)/3	4.540124…
‡15	10	0	6	4	(3, 0, − 1, 0, − 2, 0)	(322 − 199τ)/3	5.076013…
‡16	11	0	5	6	(3, 0, 0, 0, − 3, 0)	(−521 + 322τ)/6	1.568574…
‡17	9	0	5	4	(2, 0, − 2, − 1, − 2, 1)	(−377 + 233τ)/3	0.867086…
‡18	12	0	7	5	(3, − 1, − 1, − 1, − 2, − 1)	(233 − 144τ)/3	1.402975…
‡19	10	0	5	5	(2, 0, − 1, − 1, − 3, 0)	(610 − 377τ)/3	0.535889…

**Table 2:** Position of independent shell-ODs in the 6D model The Wyckoff symbol (WS) and its site symmetry (SS) are listed in the first and second columns. The atomic shell and OD type are listed in the third and fourth columns. The position of each shell-OD is given by 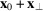. The coordinates are presented in the unit of (2 + τ)^−1/2^. The sixth column corresponds to the volume of each OD in the unit of (2 + τ)^−3/2^.

WS	SS	Shell	OD type	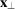	Volume
ODs at bc_0_				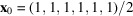	
1*a*	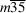	M_0_	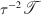	(0, 0, 0, 0, 0, 0)_⊥_	(−71 + 44τ)/6
12*a*	5*m*	B_5_	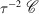	(1, 0, 0, 0, 0, 0)_⊥_	−6 + 4τ
30*a*	2*mm*	M_2_	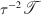	−(0, 1, 1, 0, 0, 0)_⊥_	(−32 + 21τ)/3
20*a*	3*m*	M_3_′	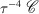	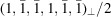	(−550 + 340τ)/3
12*a*	5*m*	M_5_′	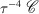	−(1, 1, 1, 1, 1, 1)_⊥_/2	−110 + 68τ
ODs at bc_1_				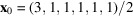	
1*b*	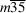	B_0_	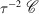	(0, 0, 0, 0, 0, 0)_⊥_	(−3 + 2τ)/6
ODs at n_0_				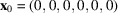	
20*a*	3*m*	M_3_	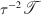	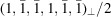	(−710 + 440τ)/3
12*a*	5*m*	M_5_	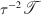	−(1, 1, 1, 1, 1, 1)_⊥_/2	(−30 + 19τ)/3
20*a*	3*m*	B_3_	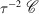	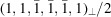	(−34 + 22τ)/3
1*a*	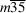	M_0_′	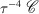	(0, 0, 0, 0, 0, 0)_⊥_	(−55 + 34τ)/6
30*a*	2*mm*	M_2_′	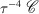	−(0, 1, 1, 0, 0, 0)_⊥_	−275 + 170τ

**Table 3:** The 24 different classes of the atomic sites for the pM+pM′ and mB clusters The volumes of the asymmetric unit of the corresponding class-ODs in the first column are listed in the second column. The frequency (in percentage) in the third column is calculated using the total volume equal to (−1229 + 763τ)/3.

Site class	Volume	Frequency
〈B_0_〉	(−3 + 2τ)/6	2.122938…
〈B_3_〉	(191 − 118τ)/3	1.294787…
〈B_3_, B_3_〉	(1877 − 1160τ)/6	0.724586…
〈B_3_, B_3_, M_2_′〉	−275 + 170τ	3.549219…
〈M_3_, B_3_〉	(−268 + 166τ)/3	10.677144…
〈M_5_, B_3_〉	(−351 + 217τ)/3	2.039153…
〈M_5_, M_5_, B_3_〉	233 − 144τ	0.167571…
〈M_5_, M_5_, M_5_, B_3_〉	(−309 + 191τ)/3	0.800222…
〈M_5_, B_3_, B_3_〉	81 − 50τ	5.304050…
〈M_5_, M_5_, B_3_, B_3_〉	(−705 + 436τ)/6	4.162092…
〈M_5_〉	(233 − 144τ)/3	0.055857…
〈M_5_, M_5_〉	(−521 + 322τ)/6	0.062450…
〈M_5_, M_5_, M_5_〉	(68 − 42τ)/3	0.765701…
〈M_3_〉	(−442 + 274τ)/3	24.124621…
〈M_0_′〉	(−55 + 34τ)/6	0.118307…
〈M_0_〉	(−71 + 44τ)/6	1.740088…
〈B_5_〉	(183 − 113τ)/3	2.916568…
〈B_5_, M_5_′〉	−110 + 68τ	1.419687…
〈M_2_, B_5_〉	(1062 − 656τ)/3	10.246586…
〈M_2_, M_2_, B_5_〉	(−933 + 577τ)/3	10.892423…
〈M_2_〉	(−302 + 187τ)/3	10.294294…
〈M_2_, M_2_〉	(382 − 236τ)/3	2.589575…
〈M_2_, M_2_, M_2_〉	(−241 + 149τ)/3	1.565923…
〈M_3_′〉	(−550 + 340τ)/3	2.366146…

**Table 4:** Subdivision of ODs for [M_3_]^1^ and [M_3_]^2^ subclasses The serial numbers in the first column represent each local configuration of the pM clusters. The volume of the subdivided subclass-OD in the asymmetric unit and frequency (in percentage) for each local configuration are given in the second and third columns, respectively. The † symbol has the same meaning as in Table 1 ▸.

No.	Volume	Frequency
[M_3_]^1^ subclass		
†1–7, 12	0	0.0
†8	(−55 + 34τ)/6	0.490400…
†9	(−55 + 34τ)/3	0.980801…
†10	(733 − 453τ)/3	2.281577…
†11	(−665 + 411τ)/3	0.892362…
†13	−377 + 233τ	0.429291…
†14	(1220 − 754τ)/3	0.176877…
[M_3_]^2^ subclass		
†1	(3029 − 1872τ)/6	1.504982…
†2	(−12831 + 7930τ)/6	0.355278…
†3	(20761 − 12831τ)/3	0.439148…
†4	(−12831 + 7930τ)/3	0.710556…
†5	(−12831 + 7930τ)/3	0.710556…
†6	(884 − 546τ)/3	41.261225…
†7	(7930 − 4901τ)/3	1.149704…
†8	(−715 + 442τ)/6	6.375209…
†9	(−2731 + 1688τ)/3	17.995279…
†10	(4419 − 2731τ)/3	11.121694…
†11	(−7150 + 4419τ)/3	6.873585…
†12	(3262 − 2016τ)/3	3.241501…
†13	(−4901 + 3029τ)/3	1.860261…
†14	(7930 − 4901τ)/3	1.149704…

## Appendix A Coordinate system

The unit vectors of the 6D lattice,  (*i* = 1, 2, …, 6), are expressed in terms of their parallel-space and perpendicular-space components, as . In the following, we use a coordinate system based on the one described in the literature (Yamamoto, 1996 ▸). The  are written using unit vectors in 3D , , , , as

with

where *a* is the edge length of the 3D icosahedral lattice. The  are written using unit vectors in 3D , , , , as

with

Note that the value of *a*′ is arbitrary because the scaling in 3D  is physically meaningless. In the present study, *a*′ is set equal to *a*.

## Appendix B Similarity transformation

The *F*-type icosahedral lattice has a similarity ratio of τ. Its similarity transformation matrix *S* is defined by  =  with (tilde means transposition)

and its inverse matrix is given by

where  (*i* = 1, 2, …, 6) are the unit vectors of the face-centred reciprocal lattice. (Note that *S* is a symmetric matrix, the determinant of which is −1.) Then the unit vectors of the icosahedral lattice are transformed by . The coordinates and Miller indices are transformed according to the same rules as for those in  and **d**_*i*_:  and . Denoting the transformation from **x** = (*x*_1_, *x*_2_, *x*_3_, *x*_4_, *x*_5_, *x*_6_) to **x**′ = (*x*_1_′, *x*_2_′, *x*_3_′, *x*_4_′, *x*_5_′, *x*_6_′) or equivalent to them under the lattice (including centring) translations as **x** → **x**′, n_1_, bc_0_ and bc_1_ are transformed cyclically: n_1_ → bc_0_ → bc_1_ → n_1_. The **E**_∥_- and **E**_⊥_-space components of the transformed unit vectors are scaled by τ and τ^−1^, respectively.

The OD shape is described by the **E**_⊥_ component of the unit vectors in the corresponding coordinate system. The *F*-type AKN tiling with edge length τ*a* is generated by a RT OD, the corners of which are given by  (*i* = 1, 2, …, 6). Its edge length is τ^−1^*a*. This means that it is obtained from such an OD located at n_0_ and bc_0_. This leads to the rule for obtaining a τ-scaled structure in the same *F*-type coordinate system. Let the OD position of a structure be **x**_*j*_ and its OD be . It is obtained from ODs scaled by τ^−1^, , located at the transformed position **x**_*j*_′. This is simply called a similarity transformation in the text.

In the inverse similarity transformation using *S*^−1^ instead of *S*, the coordinates are transformed according to a rule represented by an inverse arrow. The **E**_∥_ and **E**_⊥_ components of transformed unit vectors are scaled by τ^−1^ and τ, respectively. The unit vectors used in earlier works (Katz & Gratias, 1993 ▸; Yamamoto *et al.*, 2004*a* ▸) **d**_*i*_′ are given by the inverse transformation, so that n_0_, n_1_, bc_0_ and bc_1_ are transformed into n_0_, bc_1_, n_1_ and bc_0_, respectively. Vectors  and  are expressed by the right-hand side of equations (3) and (5) by replacing *a* with τ^−1^*a* and *a*′ with τ*a*′ in equations (4) and (6), respectively.

## References

[bb1] Bak, P. (1985*a*). *Phys. Rev. Lett.* **54**, 1517–1519. 10.1103/PhysRevLett.54.1517 10031059

[bb2] Bak, P. (1985*b*). *Phys. Rev. B*, **32**, 5764–5772. 10.1103/physrevb.32.5764 9937820

[bb3] Boissieu, M. de (2008). *Philos. Mag.* **88**, 2295–2309.

[bb4] Boissieu, M. de, Boudard, M., Hennion, B., Bellissent, R., Kycia, S., Goldman, A., Janot, C. & Audier, M. (1995). *Phys. Rev. Lett.* **75**, 89–92. 10.1103/PhysRevLett.75.89 10059122

[bb5] Boissieu, M. de, Currat, R. & Francoual, S. (2007). *Handbook of Metal Physics*, Vol. 3, *Quasicrystals*, edited by T. Fujiwara & Y. Ishii, pp. 107–169. Elsevier.

[bb6] Boissieu, M. de, Stephens, P., Boudard, M., Janot, C., Chapman, D. & Audier, M. (1994*a*). *J. Phys. Condens. Matter*, **6**, 10725–10745.

[bb7] Boissieu, M. de, Stephens, P., Boudard, M., Janot, C., Chapman, D. & Audier, M. (1994*b*). *Phys. Rev. Lett.* **72**, 3538–3541. 10.1103/PhysRevLett.72.3538 10056225

[bb8] Boudard, M., Boissieu, M. de, Janot, C., Dubors, J. M. & Dong, C. (1991). *Philos. Mag. Lett.* **64**, 197–206.

[bb9] Boudard, M., Boissieu, M. de, Janot, C., Heger, G., Beeli, C., Nissen, H.-U., Vincent, H., Ibberson, R., Audier, M. & Dubois, J. (1992). *J. Phys. Condens. Matter*, **4**, 10149–10168.

[bb10] Boudard, M., Boissieu, M. de, Letoublon, A., Hennion, B., Bellissent, R. & Janot, C. (1996). *Europhys. Lett.* **33**, 199–204.

[bb11] Cignoni, P., Callieri, M., Corsini, M., Dellepiane, M., Ganovelli, F., Ranzuglia, G., *et al.* (2008). *Eurographics Italian Chapter Conference*, Vol. 2008, pp. 129–136. Salerno, Italy.

[bb12] Cockayne, E., Phillips, R., Kan, X., Moss, S., Robertson, J., Ishimasa, T. & Mori, M. (1993). *J. Non-Cryst. Solids*, **153–154**, 140–144.

[bb13] Cornier-Quiquandon, M., Quivy, A., Lefebvre, S., Elkaim, E., Heger, G., Katz, A. & Gratias, D. (1991). *Phys. Rev. B*, **44**, 2071–2084. 10.1103/physrevb.44.2071 9999756

[bb14] Duneau, M. (2000). *Mater. Sci. Eng. A*, **294–296**, 192–198.

[bb15] Duneau, M. & Gratias, D. (2002). *Coverings of Discrete Quasiperiodic Sets: Theory and Applications to Quasicrystals*, pp. 23–62. Berlin: Springer.

[bb16] Duneau, M. & Katz, A. (1985). *Phys. Rev. Lett.* **54**, 2688–2691. 10.1103/PhysRevLett.54.2688 10031412

[bb17] Elser, V. (1985). *Phys. Rev. B*, **32**, 4892–4898. 10.1103/physrevb.32.4892 9937692

[bb18] Elser, V. (1986). *Acta Cryst.* A**42**, 36–43.

[bb19] Elser, V. (1996). *Philos. Mag. B*, **73**, 641–656.

[bb20] Elser, V. & Henley, C. L. (1985). *Phys. Rev. Lett.* **55**, 2883–2886. 10.1103/PhysRevLett.55.2883 10032264

[bb21] Francoual, S., Livet, F., de Boissieu, M., Yakhou, F., Bley, F., Létoublon, A., Caudron, R. & Gastaldi, J. (2003). *Phys. Rev. Lett.* **91**, 225501. 10.1103/PhysRevLett.91.225501 14683248

[bb22] Fujita, N., Takano, H., Yamamoto, A. & Tsai, A.-P. (2013). *Acta Cryst.* A**69**, 322–340.

[bb23] Gratias, D., Puyraimond, F., Quiquandon, M. & Katz, A. (2000). *Phys. Rev. B*, **63**, 024202. 10.1107/s0108767399006224 10927314

[bb24] Henley, C. L. (1986). *Phys. Rev. B*, **34**, 797–816. 10.1103/physrevb.34.797 9939689

[bb25] Henley, C. L. (1991). *Phys. Rev. B*, **43**, 993–1020. 10.1103/physrevb.43.993 9996293

[bb26] Janssen, T., Chapuis, G. & Boissieu, M. de (2007). *Aperiodic Crystals: from Modulated Phases to Quasicrystals.* Oxford University Press.

[bb27] Kalugin, P., Kitaev, A. & Levitov, L. (1985*a*). *JETP Lett.* **41**, 145–149.

[bb28] Kalugin, P., Kitayev, A. Y. & Levitov, L. (1985*b*). *J. Phys. Lett.* **46**, 601–607.

[bb29] Katz, A. & Gratias, D. (1993). *J. Non-Cryst. Solids*, **153–154**, 187–195.

[bb30] Kitahara, K. & Kimura, K. (2017). *Z. Kristallogr. – Cryst. Mater.*** 232**, 507–513.

[bb31] Kramer, P. & Neri, R. (1984). *Acta Cryst.* A**40**, 580–587.

[bb32] Letoublon, A., De Boissieu, M., Boudard, M., Mancini, L., Gastaldi, J., Hennion, B., Caudron, R. & Bellissent, R. (2001). *Philos. Mag. Lett.* **81**, 273–283.

[bb90] Levine, D. & Steinhardt, P. J. (1986). *Phys. Rev. B*, **34**, 596–616. 10.1103/physrevb.34.596 9939667

[bb33] Lubensky, T., Ramaswamy, S. & Toner, J. (1985). *Phys. Rev. B*, **32**, 7444–7452. 10.1103/physrevb.32.7444 9936890

[bb34] Mihalkovič, M. & Widom, M. (2020). *Phys. Rev. Res.* **2**, 013196.

[bb35] Momma, K. & Izumi, F. (2011). *J. Appl. Cryst.* **44**, 1272–1276.

[bb36] Quandt, A. & Elser, V. (2000). *Phys. Rev. B*, **61**, 9336–9344.

[bb37] Quiquandon, M. & Gratias, D. (2006). *Phys. Rev. B*, **74**, 214205.

[bb38] Quiquandon, M., Katz, A., Puyraimond, F. & Gratias, D. (1999). *Acta Cryst.* A**55**, 975–983. 10.1107/s0108767399006224 10927314

[bb39] Quiquandon, M., Portier, R. & Gratias, D. (2014). *Acta Cryst.* A**70**, 229–238. 10.1107/S2053273314004665 24815972

[bb40] Rzepski, J. D., Quivy, A., Calvayrac, Y., Corner-Quiquandon, M. & Gratias, D. (1989). *Philos. Mag. B*, **60**, 855–869.

[bb41] Shechtman, D., Blech, I., Gratias, D. & Cahn, J. W. (1984). *Phys. Rev. Lett.* **53**, 1951–1953.

[bb42] Sugiyama, K., Kaji, N., Hiraga, K. & Ishimasa, T. (1998). *Z. Kristallogr. – Cryst. Mater.*** 213**, 90–95.

[bb43] Sugiyama, K., Sun, W. & Hiraga, K. (2002). *J. Alloys Compd.* **342**, 139–142.

[bb44] Takakura, H. (2008). *Philos. Mag.* **88**, 1905–1912.

[bb45] Takakura, H., Gómez, C. P., Yamamoto, A., De Boissieu, M. & Tsai, A. P. (2007). *Nat. Mater.* **6**, 58–63. 10.1038/nmat1799 17160006

[bb46] Takakura, H. & Strzałka, R. (2017). *J. Phys. Conf. Ser.* **809**, 012002.

[bb47] Tsai, A.-P., Inoue, A. & Masumoto, T. (1987). *Jpn. J. Appl. Phys.* **26** (9A), L1505.

[bb48] Tsai, A.-P., Inoue, A. & Masumoto, T. (1988). *Jpn. J. Appl. Phys.* **27** (9A), L1587.

[bb49] Tsai, A.-P., Yokoyama, Y., Inoue, A. & Masumoto, T. (1990). *Jpn. J. Appl. Phys.* **29** (7A), L1161.

[bb50] Yamada, T. (2021). *J. Appl. Cryst.* **54**, 1252–1255. 10.1107/S1600576721005951 PMC8366420 34429725

[bb51] Yamamoto, A. (1996). *Acta Cryst.* A**52**, 509–560.

[bb52] Yamamoto, A. & Hiraga, K. (1988). *Phys. Rev. B*, **37**, 6207–6214. 10.1103/physrevb.37.6207 9943856

[bb53] Yamamoto, A., Takakura, H., Ozeki, T., Tsai, A.-P. & Ohashi, Y. (2004*a*). *J. Non-Cryst. Solids*, **334–335**, 151–155.

[bb54] Yamamoto, A., Takakura, H. & Tsai, A. (2004*b*). *Ferroelectrics*, **305**, 279–282.

[bb55] Yamamoto, A., Takakura, H. & Tsai, A. P. (2003). *Phys. Rev. B*, **68**, 094201.

[bb56] Zijlstra, E., Bose, S., Klanjšek, M., Jeglič, P. & Dolinšek, J. (2005). *Phys. Rev. B*, **72**, 174206.

